# Direct intranodal tonsil vaccination with modified vaccinia Ankara vaccine protects macaques from highly pathogenic SIVmac251

**DOI:** 10.1038/s41467-023-36907-0

**Published:** 2023-03-07

**Authors:** Jeffy G. Mattathil, Asisa Volz, Olusegun O. Onabajo, Sean Maynard, Sandra L. Bixler, Xiaoying X. Shen, Diego Vargas-Inchaustegui, Marjorie Robert-Guroff, Celia Lebranche, Georgia Tomaras, David Montefiori, Gerd Sutter, Joseph J. Mattapallil

**Affiliations:** 1 Henry M. Jackson Foundation for Military Medicine, Bethesda, MD USA; 2 Institute of Virology, University of Veterinary Medicine Hannover, Hanover, Germany; 3 Duke University School of Medicine, Durham, NC USA; 4 National Cancer Institute, Bethesda, MD USA; 5 Division of Virology, Department of Veterinary Sciences, LMU, Munich, Germany; 6 Department of Microbiology and Immunology, Uniformed Services University, Bethesda, MD USA

**Keywords:** HIV infections, Live attenuated vaccines

## Abstract

Human immunodeficiency virus (HIV) is a mucosally transmitted virus that causes immunodeficiency and AIDS. Developing efficacious vaccines to prevent infection is essential to control the epidemic. Protecting the vaginal and rectal mucosa, the primary routes of HIV entry has been a challenge given the significant compartmentalization between the mucosal and peripheral immune systems. We hypothesized that direct intranodal vaccination of mucosa associated lymphoid tissue (MALT) such as the readily accessible palatine tonsils could overcome this compartmentalization. Here we show that rhesus macaques primed with plasmid DNA encoding SIVmac251-env and gag genes followed by an intranodal tonsil MALT boost with MVA encoding the same genes protects from a repeated low dose intrarectal challenge with highly pathogenic SIVmac251; 43% (3/7) of vaccinated macaques remained uninfected after 9 challenges as compared to the unvaccinated control (0/6) animals. One vaccinated animal remained free of infection even after 22 challenges. Vaccination was associated with a ~2 log decrease in acute viremia that inversely correlated with anamnestic immune responses. Our results suggest that a combination of systemic and intranodal tonsil MALT vaccination could induce robust adaptive and innate immune responses leading to protection from mucosal infection with highly pathogenic HIV and rapidly control viral breakthroughs.

## Abstract (web summary)

Mucosal surfaces are a primary route of HIV entry, yet the compartmentalisation between mucosal and peripheral immune systems remain a challenge for HIV vaccine candidates. Authors utilise a combination of intranodal tonsil MALT and systemic vaccination in the rhesus macaque model to explore immune responses and protection from highly pathogenic simian homologue of HIV.

## Introduction

Human immunodeficiency virus (HIV) infection is characterized by progressive loss of CD4 T cells leading to immunodeficiency and AIDS. The advent of highly active antiretroviral therapy (HAART) has had a significant impact on people living with HIV leading to better long-term outcomes. There are, however, a significant number of new HIV infections that are reported worldwide every year. According to the UNAIDS 2021 report, a total of 1.5 million people were newly infected with HIV in 2020 suggesting that HIV transmission continues to be a major public health concern. The highest rates of new infections are reported each year in Africa and other resource limited countries^1^. As such, there continues to be an urgent need to develop highly efficacious vaccines and immunization strategies that can prevent HIV transmission and the emergence of new infections especially in resource limited settings where transmission rates remain quite high^2,3^.

HIV vaccine development, however, remains a work in progress given the difficulty of generating immunogens that can induce potent and broad neutralizing antibody (nAb) responses in vivo. Compounding the challenge is the mucosal nature of HIV acquisition and transmission^1,4^. HIV vaccines that can prevent acquisition, and at the same time effectively control breakthrough infections are essential to contain HIV transmission.

There are currently no FDA approved vaccines against HIV. Numerous vaccine candidates have been tested in Phase 3 clinical trials. With the exception of one candidate, most have not been successful in meeting their primary end points. The R144 trial was the only successful HIV vaccine trial to date but exhibited a modest efficacy of 31%. A follow-up trial (HVTN702) failed to recapitulate these findings. Interestingly, the vaccine efficacy in the RV144 trial was associated with non-nAb gp120 V1V2 specific responses instead of nAbs suggesting that indirect mechanisms rather than direct neutralization of HIV likely contributed to the protection seen in the trial.

Though mucosal sites remain the primary target for the induction of immune responses by HIV vaccine, the highly compartmentalized nature of the mucosal vs systemic immune systems imposes a major constraint on the durable induction of mucosal immune responses by systemically delivered vaccines. Most vaccine candidates in advanced clinical trials rely on systemic delivery of vaccine immunogens. Numerous preclinical studies have, however, examined mucosal vaccination using SIV or SHIV challenge models with the intent of inducing immune responses not only at the site of vaccination but also at distal mucosal sites. The mucosa associated lymphoid tissue (MALT) includes the lymphoid tissue in the nasopharynx, gastrointestinal tract and the cervico-vaginal mucosa with immune cells continuously trafficking between these sites as a part of the common mucosal immune system^5–7^. Barnett et al.^8^ demonstrated that a combination of intramuscular (IM) and intranasal (IN) vaccination with HIV DNA and envelope proteins induced potent immune responses in the vaginal mucosa that protected rhesus macaques from intravaginal challenge SHIV_SF162P4_ challenge. Zhou et al.^9^ showed that a combination of oral and IN immunization with replication competent adenovirus-5 recombinant vaccine induced peripheral and mucosal immune responses correlated with control of chronic SIVmac251 infection. More recently, needle free sublingual vaccination with MVA and gp120 was found to induce HIV specific immune responses against multiple HIV-1 clades that significantly delayed acquisition of SHIV_SF162P3_ infection which correlated with V2 specific antibody responses^10^. Others have reported similar results using an oral challenge model of neonatal macaques that were immunized using a combination of IM and sublingual routes^11^. Manrique et al.^12^ reported that mucosal vaccination was associated with early and persistent suppression of SIVmac251 viremia following intravaginal challenge.

Numerous preclinical and clinical studies have demonstrated the efficacy of intranodal immunization against malignancies^13–16^. Lambert et al.^17^ and Bedrosian et al.^18^ compared intranodal immunization with dendritic cell vaccines to other routes such as intradermal and found that intranodal immunization was more effective at inducing tumor specific CD8 T cell and DTH responses. No study to date has examined direct intranodal immunization of the mucosal tissue to determine HIV vaccine efficacy. We hypothesized that direct immunization of the tonsils with MVA encoding SIVmac251-env and gag genes would effectively boost systemically primed immune responses leading to better protection from a low dose mucosal challenge and lead to a better control of viremia in cases of breakthrough infections. Palatine tonsils are an easily accessible organized lymphoid tissue that is highly enriched in B cell follicles along with T cells and dendritic cells. Rhesus macaques were primed with plasmid DNA encoding SIVmac251-env and gag genes intramuscularly by electroporation followed by direct vaccination of the tonsils with MVA encoding the same genes. All macaques were challenged intrarectally with a repeated low dose of SIVmac251. Our results demonstrated a significant level of protection from acquisition, and ~2 log reduction in acute plasma viremia that correlated with B, T and NK cell responses. Our findings suggest that a combination of systemic and intranodal tonsil MALT vaccination has the potential to induce robust and broad protective immune responses against HIV infection.

## Results

### Tonsils are readily infected with MVA

**Fig. 1:**
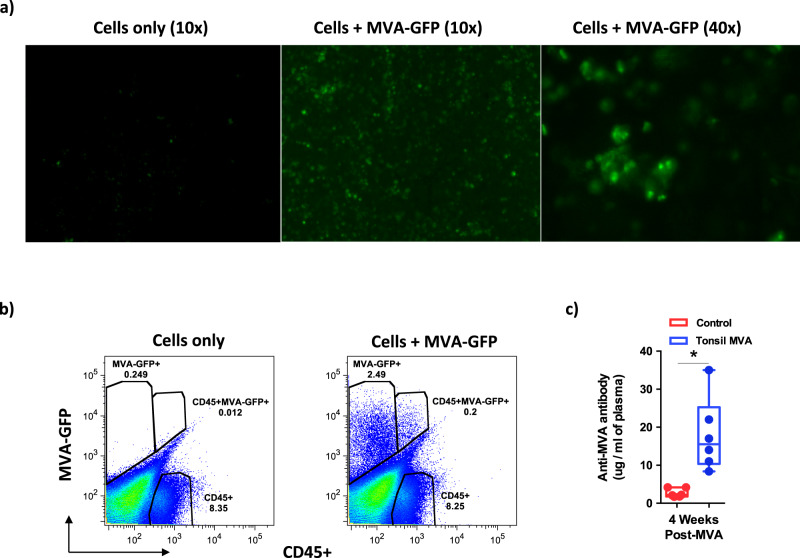
Tonsils are readily infected by MVA. Tonsil cells were co-cultured with MVA-GFP at a MOI = 1.0 for 24 h and were examined by (**a**) fluorescent microscopy and (**b**) labeled with anti-CD45 and examined flow cytometry. Tonsils were collected from a miscellaneous rhesus macaque at necropsy. As such, the co-culturing experiment was only performed once. (**c**) Anti-MVA antibody levels in the plasma that was collected 4 weeks after tonsil vaccination with MVA (*n* = 6) as compared to control animals (*n* = 6). Box plots show minima, 25% percentile, median, 75 percentile and maxima. Statistical analysis was performed using two-tailed Mann–Whitney *U* test (*P* = 0.0011). A *P* < 0.05 (*) was considered significant. * indicate *P* < 0.05. Error bars represent standard error.

To determine if MVA effectively infected and replicated in the tonsils, we isolated cells from the tonsils of a rhesus macaque and co-cultured these cells with MVA encoding GFP at an MOI = 1 and examined them by fluorescent microscopy and flow cytometry at 24 h PI (Supplementary Fig. 1). Our results (Fig. 1a, b) showed that MVA readily infected both CD45^−^ and CD45^+^ cells suggesting that tonsils could readily support MVA infection. To assess if in vitro infection translated to in vivo replication of MVA, we examined MVA specific binding antibody (bAb) levels in the plasma of rhesus macaques that received the tonsil MVA vaccine at 4 weeks post vaccination (Fig. 1c). Our results demonstrated that tonsil vaccination induced significant levels of anti-MVA antibody responses suggesting that intranodal MVA vaccination could be a highly effective approach to induce immune responses.

### Tonsil vaccination with MVA prevented acquisition in 3/7 animals after 9 repeated low dose IR challenges with SIVmac251

**Fig. 2:**
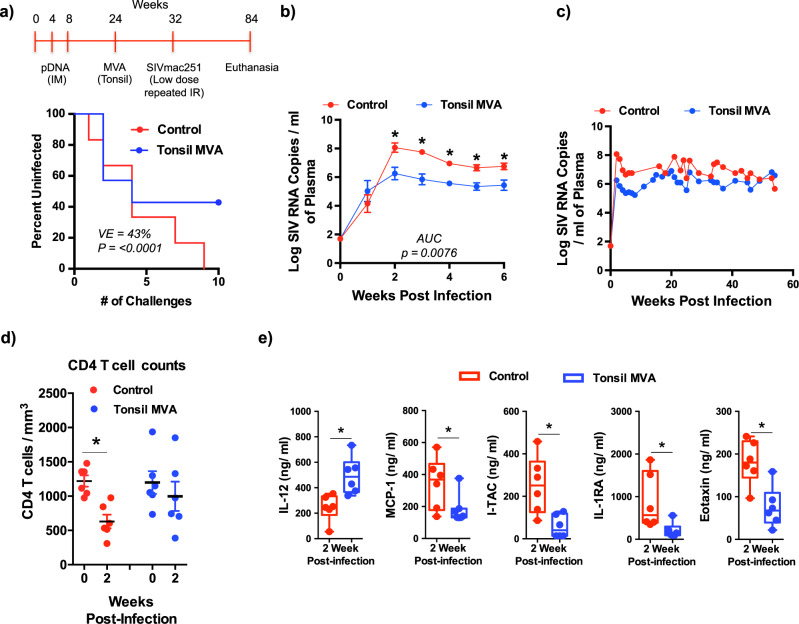
Tonsil vaccination with MVA prevents SIVmac251 acquisition and is associated with control of acute viremia. (**a**) Study design and percentage of uninfected animals after repeated low dose intrarectal challenge with SIVmac251 in tonsil vaccinated (*n* = 6) and control (*n* = 6) groups. Statistical analysis was performed using one-tailed Fishers Exact Test as described in ref. ^101^ and a *P* < 0.05 (*) was considered significant. Kinetics of plasma viral loads (Limit of detection is 50 copies/ml of plasma) (**b**) during 1^st^ 6 weeks after infection and (**c**) over a period of 52 weeks in control (*n* = 6) and tonsil vaccinated (*n* = 6) animals. Area Under Curve (AUC) was determined using Prism followed by Mann–Whitney *U* test with error bars represent standard error. (**d**) Absolute CD4 T cells counts at 0 and 2 weeks post infection is control (*n* = 6; *P* = 0.0043) and tonsil vaccinated (*n* = 6; *P* = ns) animals. Data are presented as mean values + /– standard error. Statistical analysis was performed using two-tailed Mann–Whitney *U* test and a *P* < 0.05 (*) was considered significant. Error bars represent standard error. (**e**) Plasma levels of IL-12 (*P* = 0.0022), MCP-1 (*P* = 0.0332), I-TAC (*P* = 0.0087), IL-1RA (*P* = 0.0152), and Eotaxin (*P* = 0.0043) at 2 weeks post infection in control (*n* = 6) and tonsil vaccinated (*n* = 6) animals. Unbiased cytokine levels were determined using a 29-plex Monkey cytokine kit. Box plots show minima, 25% percentile, median, 75 percentile and maxima. Statistical analysis was performed using two-tailed Mann–Whitney *U* test and a *P* < 0.05 (*) was considered significant. * indicate *P* < 0.05. Error bars represent standard error.

Next, we evaluated if the MVA SIV-env and gag vaccine directly delivered to the tonsils was effective in preventing acquisition of mucosal SIV infection. The MVA vaccine was directly injected into the tonsils, 50 µl on each side. The animals were monitored over a period of 2 weeks and we did not observe any apparent inflammation on the tonsils following vaccination. To determine vaccine efficacy, rhesus macaques were challenged with a low dose of highly pathogenic SIVmac251 by the IR route at weekly intervals until all the rhesus macaques in the unvaccinated control group had detectable plasma viremia (≥50 SIV RNA copies/ml). All 6/6 control animals were infected by the 9th low dose IR challenge whereas only 4/7 vaccinated animals were infected by the 9th challenge demonstrating a VE = 43% (Fig. 2a; *P* < 0.0001). We continued to challenge the remaining 3/7 uninfected vaccinated animals at weekly intervals until the onset of infection; of these 3 animals, one became infected after the 11th and one after 19th challenge whereas one remained free of infection even after 22 challenges at which time challenges were stopped due to lack of additional challenge stock.

### Vaccination of the tonsils with MVA was associated with a ~ 2 log reduction in acute plasma viremia

Protective immune responses play a key role in control of viral infection. To determine if vaccination was associated with better control of SIV infection in cases of breakthrough, we examined the kinetics of plasma viremia in vaccinated animals (*n* = 6) that became infected and compared them to control animals (*n* = 6). Our results showed that acute plasma viremia in tonsil vaccinated animals was ~2 log lower than that of the control animals (Fig. 2b); peak viremia was ~6 logs of SIV RNA copies/ml of plasma in vaccinated animals as compared to ~8 logs of SIV RNA copies/ml of plasma in control animals suggesting the anamnestic vaccine induced immune responses were highly effective in suppressing acute viral replication. Post-acute plasma viral loads at 8 weeks PI, however, did not differ significantly over the course of the next 44 weeks indicating loss of viral control by vaccine induced immune responses (Fig. 2c and Supplementary Fig. 2). This is not surprising given the pathogenicity and potential of SIVmac251 to generate quasispecies during replication that readily escape immune control.

Suppression of acute viremia was associated with better preservation of CD4 T cell counts in vaccinated animals in contrast to a significant decrease in control animals (Fig. 2d). To determine if the decrease in plasma viral loads was accompanied by lower inflammatory responses, we examined the plasma levels of 29 pro- and anti-inflammatory cytokines simultaneously at 2 weeks PI using a 29-plex cytokine kit in collaboration with the Duke Human Vaccine Institute. We observed a surprisingly significant increase in plasma IL-12 levels in vaccinated animals at 2 weeks PI as compared to control animals (Fig. 2e). In contrast, there was a significant decrease in the plasma levels of MCP-1, I-TAC, IL-1RA, and Eotaxin in vaccinated animals (Fig. 2e) suggesting the lower viremia following vaccination significantly reduced an acute inflammatory response and likely immune activation.

### Significant anamnestic increase anti-SIVmac251.6 nAb responses negatively correlated with plasma viremia

Studies have reported that passive transfer of nAb effectively protected rhesus macaques from mucosal infection^19–21^. To determine if tonsil vaccination and the protection we observed was associated with the induction of SIVmac251 specific nAb responses, we examined levels of SIVmac251 gp120 specific bAb and nAb titres prior to infection and at 2 weeks PI.

**Fig. 3:**
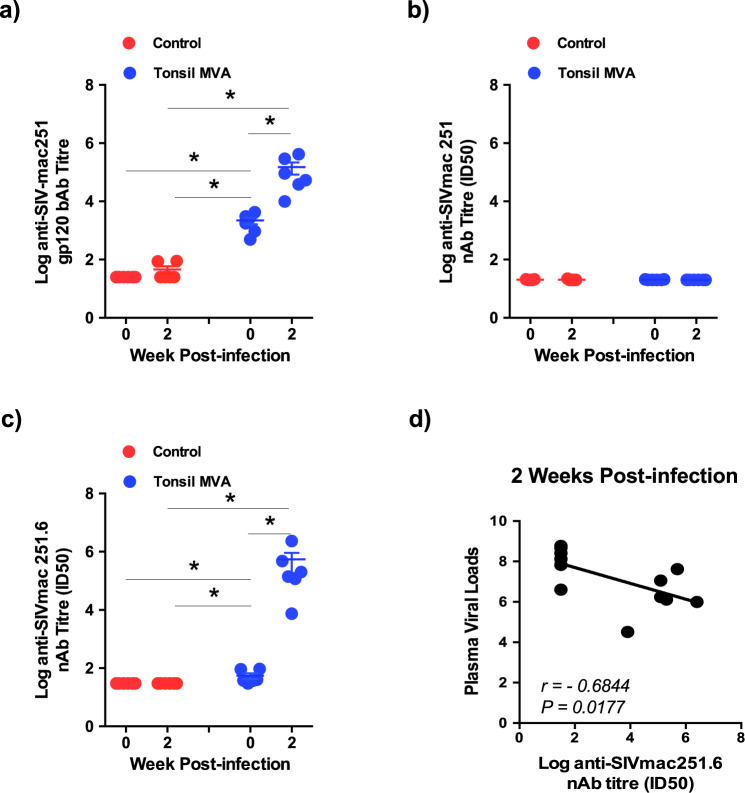
Tonsil vaccination induced significant levels of SIVmac251.6 neutralizing antibodies that inversely correlated with plasma viral loads. SIVmac251 specific (**a**) gp120 binding antibody (week 0 control vs week 2 control *P* = ns; week 0 control vs week 0 tonsil MVA *P* = 0.0011; week 2 control vs week 0 tonsil MVA *P* = 0.0011; week 2 control vs week 2 tonsil MVA *P* = 0.0011; week 0 tonsil MVA vs week 2 tonsil MVA *P* = 0.0011). Data are presented as mean values + /– standard error, Statistical analysis was performed using one-tailed Mann–Whitney *U* test and a *P* < 0.05 (*) was considered significant. Error bars represent standard error. (**b**) SIVmac251 specific neutralizing antibody responses in control (*n* = 6) and tonsil vaccinated (*n* = 6) animals at 0 and 2 weeks post infection. (**c**) SIVmac251.6 specific neutralizing antibody levels in the plasma of control (*n* = 6) and tonsil vaccinated (*n* = 6) animals at 0 and 2 weeks post infection (week 0 control vs week 2 control *P* = ns; week 0 control vs week 0 tonsil MVA *P* = 0.0076; week 2 control vs week 0 tonsil MVA *P* = 0.0076; week 2 control vs week 2 tonsil MVA *P* = 0.0011; week 0 tonsil MVA vs week 2 tonsil MVA *P* = 0.0011). Data are presented as mean values + /– standard error. Statistical analysis was performed using one-tailed Mann–Whitney *U* test and a *P* < 0.05 (*) was considered significant. * indicate *P* < 0.05. Error bars represent standard error. (**d**) Correlation between plasma viral loads and anti-SIVmac251.6 neutralizing antibody levels at 2 weeks post infection was determined using Spearman’s rank test and a *P* < 0.05 was considered significant.

Vaccination induced significant levels of SIVmac251 gp120 bAb responses prior to infection that was significantly boosted at 2 weeks PI suggesting a strong anamnestic response following infection (Fig. 3a). We did not detect nAb responses against the SIVmac251 challenge virus suggesting that tonsil vaccination failed to induce tier 1B SIVmac251 specific nAb responses (Fig. 3b) that was not surprising given the neutralization resistant phenotype of the challenge strain. In contrast, we observed detectable levels of tier 2A SIVmac251.6 specific nAb response prior to challenge that was significantly boosted at 2 weeks PI (Fig. 3c). SIVmac251.6 specific nAb response was found to negatively correlate (*r* = *−*0.6844*, P* = 0.0177) with peak plasma viral loads at 2 weeks PI (Fig. 3d) suggesting a role of tier 2 A specific responses in acute viral control.

### Tonsil vaccination induced high levels of SIVmac251, 239 and SIVsmE660 V1/V2 specific antibody responses that were significantly boosted after infection

The R144 clinical trial demonstrated that levels of HIV gp120 V1V2 specific non-nAb responses significantly correlated with protection from HIV infection in vaccinated individuals^22,23^. Numerous studies were found to confirm this correlation of V1V2 specific non-nAb responses with protection or control of either SIV or SHIV infections^24^. Vaccination with Aventis Pasteur’s canarypox vector (ALVAC) encoding SIV gp120 with alum alone or after a DNA prime induced V2 specific responses that was associated with either delayed onset or risk of acquisition of SIVmac251 in rhesus macaques^25–27^ whereas an HIV clade B/C ALVAC/gp120 vaccine with a high dose of alum induced V2 specific IgG responses in serum that correlated with a reduced risk for SHIV-C acquisition in rhesus macaques.

**Fig. 4:**
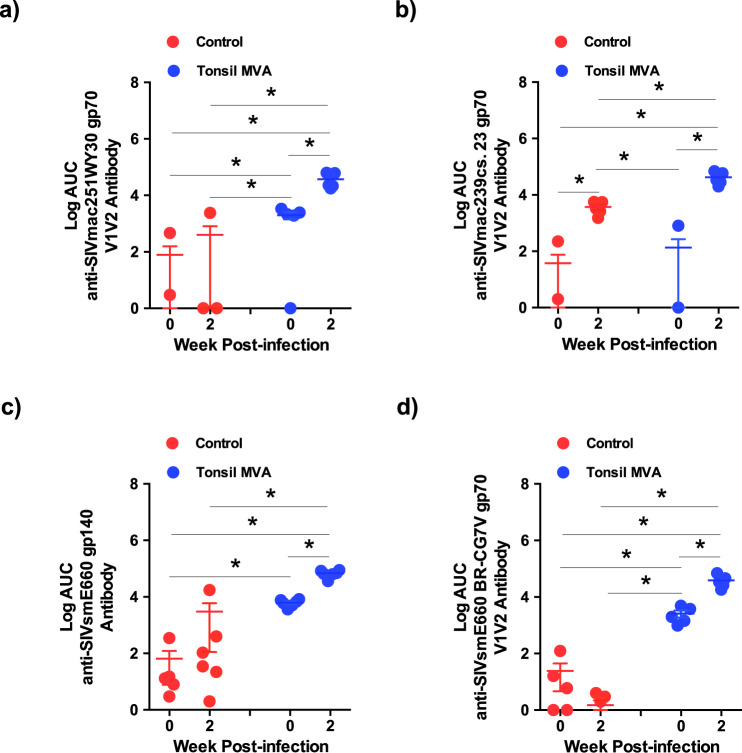
Tonsil vaccination induced significant levels of V1V2 specific antibodies against homologous and heterologous strains of SIV. (**a**) SIVmac251-WY30 gp70 V1V2 antibody (week 0 control vs week 2 control *P* = ns; week 0 control vs week 0 tonsil MVA *P* = 0.0087; week 2 control vs week 0 tonsil MVA *P* = 0.0216; week 2 control vs week 2 tonsil MVA *P* = 0.0022; week 0 tonsil MVA vs week 2 tonsil MVA *P* = 0.0040). (**b**) SIVmac239-cs.23 gp70 (week 0 control vs week 2 control *P* = 0.0022; week 0 control vs week 0 tonsil MVA *P* = ns; week 2 control vs week 0 tonsil MVA *P* = 0.0022; week 2 control vs week 2 tonsil MVA *P* = 0.0040; week 0 tonsil MVA vs week 2 tonsil MVA *P* = 0.0022). (**c**) SIVsmE660-gp140 (week 0 control vs week 2 control *P* = ns; week 0 control vs week 0 tonsil MVA *P* = 0.0011; week 2 control vs week 0 tonsil MVA *P* = 0.0325; week 2 control vs week 2 tonsil MVA *P* = 0.0011; week 0 tonsil MVA vs week 2 tonsil MVA *P* = 0.0011), and (**d**) SIVsmE660-BR-CG7V gp70 (week 0 control vs week 2 control *P* = ns; week 0 control vs week 0 tonsil MVA *P* = 0.0022; week 2 control vs week 0 tonsil MVA *P* = 0.0022; week 2 control vs week 2 tonsil MVA *P* = 0.0022; week 0 tonsil MVA vs week 2 tonsil MVA *P* = 0.0040). specific antibody responses in control (*n* = 6) and tonsil vaccinated (*n* = 6) animals at 0 and 2 weeks post infection. Data are presented as mean values + /– standard error. Statistical analysis was performed using one-tailed Mann–Whitney *U* test and a *P* < 0.05 (*) was considered significant. * indicate *P* < 0.05. Error bars represent standard error.

To determine if tonsil vaccination with MVA encoding SIV-env and gag induced SIVgp120 V1V2 specific non-nAb responses, we examined SIVmac251-WY30 gp70 and SIVmac239-CS.23 gp70 V1V2 specific antibody levels in vaccinated animals prior to infection and at 2 weeks PI and compared them to control animals. Our results demonstrated that tonsil vaccination induced significant levels of tier 1B SIVmac251-WY30 gp70 V1V2 specific antibody responses prior to infection as compared to controls that were significantly boosted at 2 weeks PI (Fig. 4a). Interestingly, we did not observe a significant induction of tier 2 SIVmac239-CS23 V1V2 specific antibody responses prior to infection in either the control or vaccinated groups of animals (Fig. 4b). SIV infection was, however, associated with a significant increase in SIVmac239-CS.23 gp70 V1V2 specific antibody responses at 2 weeks PI in both control and vaccinated animals with the responses being significantly higher in vaccinated animals after infection as compared to the control animals suggesting that tonsil vaccination likely primed tier 2 SIVmac239-CS.23 non-neutralizing V1V2 antibody responses prior to infection albeit at low levels that were boosted anamnestically after infection. Previous studies have shown that vaccines could induce V2 or V1V2 specific antibody responses in NHP models that were associated with protection^28–31^.

Tonsil vaccinated animal’s demonstrated significant levels of SIVsmE660 bAb and V1V2 responses prior to infection as compared to control animals (Fig. 4c, d). SIV infection was associated with a significant anamnestic increase in V1V2 specific responses suggesting that tonsil vaccination was highly effective in inducing non-nAb responses to heterologous SIV isolates. Singh et al.^32^ reported that induction of V1V2 specific binding antibodies correlated with decreased risk of SIVsmE660 acquisition.

### Significant amplification of ADCC and NK cell responses after SIV infection inversely correlated with acute plasma viremia

**Fig. 5:**
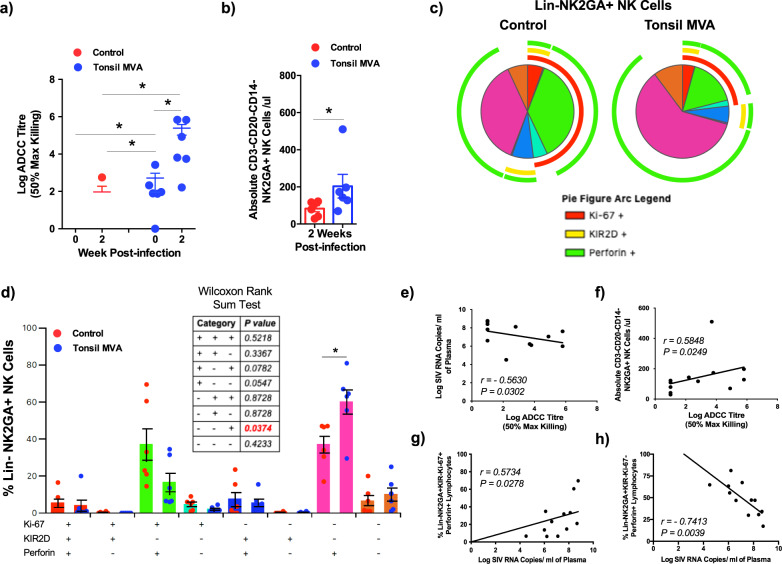
Tonsil vaccination induced high levels of ADCC and NK cell responses that inversely correlated with acute plasma viremia. (**a**) Log ADCC titre (50% max killing) in control (*n* = 6) and tonsil vaccinated (*n* = 6) animals at 0 and 2 weeks post infection. Data are presented as mean values + /– standard error. Statistical analysis was performed using Mann–Whitney *U* test and a *P* < 0.05 (*) was considered significant. Error bars represent standard error. (**b**) Absolute numbers of NK cells in peripheral blood of control (*n* = 6) and tonsil vaccinated (*n* = 6) animals 2 weeks post infection (*P* = 0.0130). Data are presented as mean values + /– standard error. Statistical analysis was performed using One-tailed Mann–Whitney *U* test and a *P* < 0.05 (*) was considered significant. Error bars represent standard error. (**c**) Qualitative analysis of Ki-67, KIR2D and perforin expression in Lin^−^NKG2A^+^ NK cells in peripheral blood of control (*n* = 6) and tonsil vaccinated (*n* = 6) animals at 2 weeks post infection. (**d**) Frequency of Lin^−^NKG2A^+^ NK cells in peripheral blood of control (*n* = 6) and tonsil vaccinated (*n* = 6) animals that express Ki-67, KIR2D and perforin at 2 weeks post infection. Statistical analysis was performed using two-sided Wilcoxon rank sum test and a *P* < 0.05 (*) was considered significant. *indicate *P* < 0.05. Error bars represent standard error. Correlation between (**e**) plasma viral loads and log ADCC titre at 2 week post infection, (**f**) absolute numbers of NK cells and log ADCC titre, (**g**) frequency of LIN^−^NKG2A^+^KIR2D^−^Ki-67^+^Perforin^+^ NK cells and plasma viral loads at 2 weeks post infection, and (**h**) the frequency of LIN^−^NKG2A^+^KIR2D^−^Ki-67^−^Perforin^+^ NK cells and plasma viral loads at 2 weeks post infection in control (*n* = 6) and tonsil vaccinated (*n* = 6) animals. Correlations for (**e**–**h**) were determined using Spearman’s rank test and a *P* < 0.05 was considered significant.

The R144 trial demonstrated that ADCC responses inversely correlated with risk for HIV infection^22,33,34^. To determine if ADCC responses played a role in suppressing acute viremia in tonsil vaccinated animals, we examined the 50% Max Killing ADCC titres in vaccinated animals at 2 weeks PI and compared them to control animals (Fig. 5a). Our results demonstrated that vaccinated animals had significantly higher levels of ADCC responses prior to infection that were boosted significantly at 2 weeks PI. ADCC titres had a significant negative correlation with plasma viral loads (Fig. 5e, *r* = −0.5630, *P* = 0.0302) suggesting that higher ADCC titres played a role in the acute suppression of viremia after infection.

NK cells play a critical role in mediating ADCC responses. To determine if higher ADCC responses accompanied changes in NK cells, we examined the absolute numbers of CD3^−^CD20^−^CD14^−^NKG2A^+^ NK cells in peripheral blood of tonsil vaccinated animals and compared them to control animals. Our results showed that tonsil vaccinated animals displayed significantly higher numbers of NK cells as compared to control animals after SIV infection (Fig. 5b) that had a significant positive correlation with ADCC titres (Fig. 5f, *r* = 0.5848*, P* = 0.0249) suggesting that NK cells likely contributed to the increased ADCC responses.

Next we sought to determine if higher numbers of NK cells in tonsil vaccinated animals translated to qualitative differences in the nature of NK cells as compared to control animals. To address this question, we examined the levels of Ki-67, KIR2D, and perforin levels in CD3^−^CD20^−^CD14^−^NKG2A^+^ NK cells of vaccinated animals at 2 weeks PI and compared them to control animals (Fig. 5c, d and Supplementary Fig. 3). Interestingly, NK cells from control animals expressed higher levels of Ki-67 (red arc in Fig. 5c) that were accompanied by higher frequencies of Ki-67^+^Perforin^+^ cells as compared to tonsil vaccinated animals though these differences were not significant (Fig. 5d). Rather surprisingly, we noted a significant positive correlation between CD3^−^CD20^−^CD14^−^NKG2A^+^KI-67^+^KIR2D^−^Perforin^+^ NK cells and plasma viral loads (Fig. 5g, *r* = 0.5734, *P* = 0.0278) suggesting that even though the absolute numbers of NK cells were significantly lower in control animals as compared to vaccinated animals, changes in NK cell subsets were associated with immune activation and increased viral replication. In contrast to higher frequencies CD3^−^CD20^−^CD14^−^NKG2A^+^KI-67^+^KIR2D^−^Perforin^+^ NK cells in control animals, vaccinated animals had significantly higher frequencies of CD3^−^CD20^−^CD14^−^NKG2A^+^KI-67^−^KIR2D^−^Perforin^+^ NK cells that had a significant inverse correlation with plasma viral loads (Fig. 5h, *r* = −0.7413*, P* = 0.0039) suggesting that vaccinated animals displayed significantly higher numbers of NK cells that were capable of cytolytic function that in turn likely contributed to the suppression of acute viremia following SIV infection.

### Tonsil vaccination induced significantly higher poly-functional T cell responses after SIV infection

**Fig. 6:**
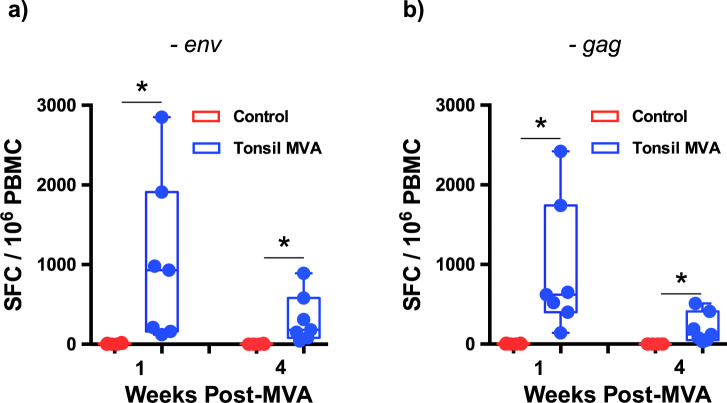
Tonsil vaccination induced high levels of T cell ELISPOT responses prior to challenge. SIVmac239 (**a**) env (week 1 control vs week 1 post-MVA *P* = 0.0006; week 4 control vs week 4 post-MVA *P* = 0.0006) and (**b**) gag (week 1 control vs week 1 post-MVA *P* = 0.0006; week 4 control vs week 4 post-MVA *P* = 0.0006) specific T cell responses in PBMC by ELISPOT assay at 1 and 4 weeks after tonsil vaccination (*n* = 7) prior to challenge with SIVmac251 as compared to control (*n* = 6) animals. Data arereported as spot forming counts (SFC)/10^6^ cells. Box plots show minima, 25% percentile, median, 75 percentile and maxima. Statistical analysis was performed using one-tailed Mann–Whitney *U* test and a *P* < 0.05 (*) was considered significant. * indicate *P* < 0.05. Error bars represent standard error.

Studies have clearly established a critical role of T cell responses in the control of HIV and SIV infections^35–40^. To determine if tonsil vaccination induced SIV specific T cell responses, we examined total SIVmac239-env and gag specific T cell responses using ELISPOT assay prior to and after SIV infection in vaccinated animals and compared them to control animals. Vaccinated animals were found to have significantly higher levels of IFNγ responses prior to infection at 1 and 4 weeks after tonsil vaccination as compared to control animals (Fig. 6a, b).

**Fig. 7:**
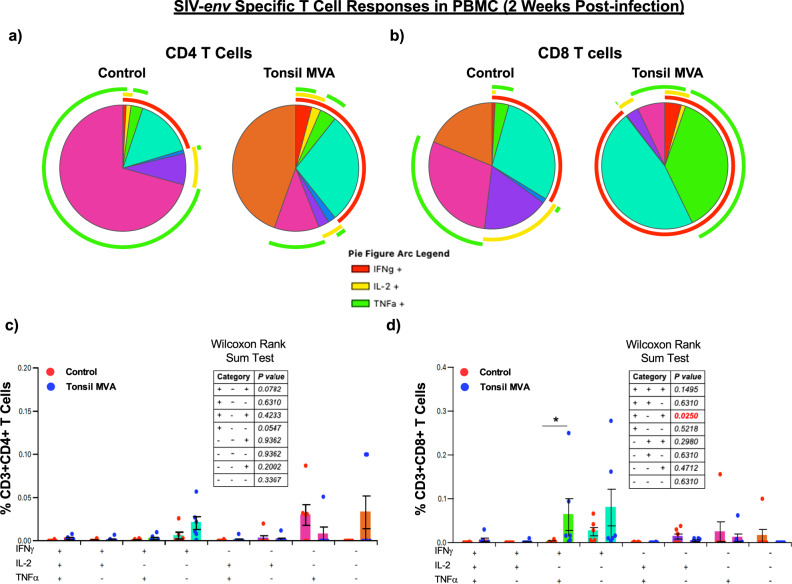
Tonsil vaccination induced significant levels of SIVmac239-env specific poly-functional CD8 T cell responses in peripheral blood. Qualitative analysis of SIVmac239-env specific IFNγ, IL-2, and TNFα expression in peripheral blood (**a**) CD4 and (**b**) CD8 T cells of control (*n* = 6) and tonsil vaccinated (*n* = 6) animals at 2 weeks post infection. Frequency of (**c**) CD3^+^CD4^+^ T cells and (**d**) CD3^+^CD8^+^ T cells in peripheral blood of control (*n* = 6) and tonsil vaccinated (*n* = 6) animals that express IFNγ, IL-2, and TNFα at 2 weeks post infection. Statistical analysis was performed using two-sided Wilcoxon rank sum test in SPICE software and a *P* < 0.05 (*) was considered significant. * indicate *P* < 0.05. Error bars represent standard error.

**Fig. 8:**
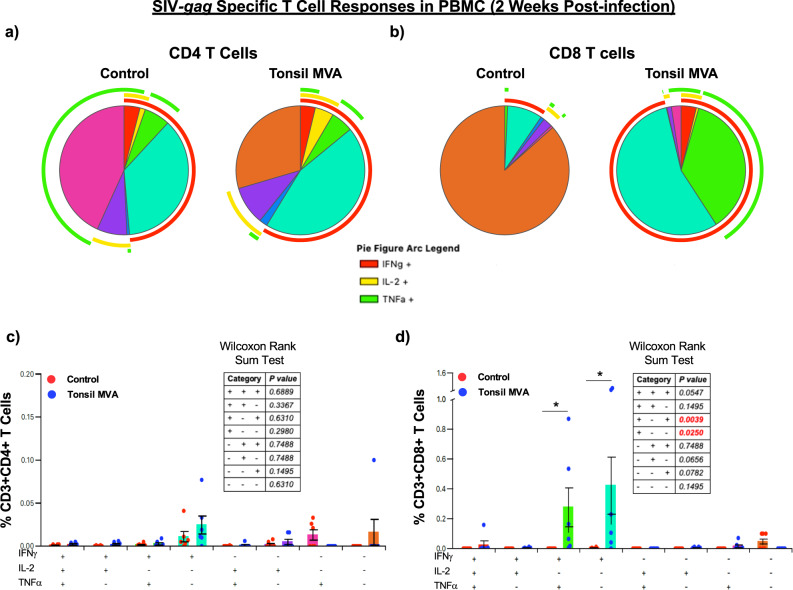
Tonsil vaccination induced significant levels of SIVmac239-gag specific poly-functional CD8 T cell responses in peripheral blood. Qualitative analysis of SIVmac239-gag specific IFNγ, IL-2, and TNFα expression in peripheral blood (**a**) CD4 and (**b**) CD8 T cells of control (*n* = 6) and tonsil vaccinated (*n* = 6) animals at 2 weeks post infection. Frequency of (**c**) CD3^+^CD4^+^ T cells and (**d**) CD3^+^CD8^+^ T cells in peripheral blood of control (*n* = 6) and tonsil vaccinated (*n* = 6) animals that express IFNγ, IL-2, and TNFα at 2 weeks post infection. Statistical analysis was performed using two-sided Wilcoxon rank sum test in SPICE software and a *P* < 0.05 (*) was considered significant. * indicate *P* < 0.05. Error bars represent standard error.

Next we sought to determine if there were qualitative differences in the nature of CD4 and CD8 T cell responses between tonsil vaccine induced anamnestic responses and immune responses in control animals. To address this question, we examined the expression of IFNγ, IL-2 and TNFα expression in SIVmac239-env and gag specific peripheral blood CD4 and CD8 T cells at 2 weeks PI using flow cytometry. Our results demonstrated that tonsil vaccination induced poly-functional anamnestic responses; SIV-env specific CD4 T cell responses was characterized by higher levels of IFNγ in vaccinated animals as compared to control animals (red arc in Fig. 7a, c). In contrast, there was a significant difference in the magnitude of poly-functional SIV-env specific CD8 T cell responses (IFNγ^+^IL-2^−^TNFα^+^) in tonsil vaccinated animals as compared to the control animals (Fig. 7b, d). A similar trend was observed with respect SIV-gag specific CD4 and CD8 T cell responses; CD8 T cell responses were dominated by IFNγ responses with significantly higher levels of SIV-gag specific IFNγ^+^IL-2^−^TNFα^+^ and IFNγ^+^IL-2^−^TNFα^−^CD8 T cells as compared to control animals (Fig. 8a–d).

We were unable to sample the rectal mucosa during the early stages of infection due to the repeated low dose nature of the challenge and the potential for inducing inflammation and immune activation in the mucosa. However, we sampled both the control and vaccinated animals at 4 weeks after infection was confirmed, and used isolated cells to determine SIV-env and gag specific T cell responses by flow cytometry. We did not observe a significant difference in either the magnitude or poly-functionality of SIV-env or gag specific CD4 or CD8 T cell responses between the two groups though tonsil vaccinated animals tended to have higher levels of IL-2^+^ responses (yellow arc) in contrast to TNFα^+^ responses (green arc) in control animals (Suppl. Figs. 4 and 5). The lack of a significant difference in the magnitude of responses was not surprising given the sampling at 4 week PI and is in line with some earlier studies showing that T cell responses appear to wane with a decrease in antigen load that usually peaks at 2 weeks PI^41^.

## Discussion

Direct vaccination of the palatine tonsils has been an underexplored area of research. However, the easy and ready access to an organized lymphoid tissue that is enriched for all the key immune cells critical for the generation of potent immune responses offers an attractive target. In fact, macaque tonsils like the human tonsils are highly enriched in B cell follicles, T cells and Dendritic cells and effective infection with MVA encoding GFP demonstrated that tonsillar cells could readily support replication of the MVA vaccine encoding SIV genes. Given that tonsils are a part of the common mucosal immune system where immune cells traffic between different mucosal sites, we hypothesized that vaccinating the tonsils with an MVA vaccine encoding SIVmac239-env and gag genes could protect from acquisition of SIV infection following a low dose rectal challenge and in cases of breakthroughs lead to better control of SIV infection and replication.

As a proof of concept, macaques were vaccinated with a single dose of MVA vaccine encoding SIVmac239-env and gag genes by direct intranodal injection of both the palatine tonsils and monitored for over 2 weeks for signs of inflammation. Tonsil cells were readily infectable with MVA in vitro (Fig. 1a, b) and vaccination induced significant levels of MVA specific bAb at 4 weeks post vaccination (Fig. 1c), suggesting that tonsils could be effectively immunized with MVA encoding immunogens. We did not observe any overt signs of inflammation on the tonsils during this period nor was there any change in the eating and drinking behavior of animals that received the vaccine suggesting that vaccinating the tonsils could be explored as a viable approach for further experimentation.

Though we directly injected the vaccine into the tonsils in this proof-of-concept study, the tonsils are highly amenable to non-traumatic application of immunogens. Tonsils consist of ~20 to 30 tonsillar crypts in macaques^42^, and ~12 to 15 in humans^43,44^. The crypts, unlike the rest of the tonsil surface, are lined by epithelium that is fragmented and discontinuous with constant exchange between the lumen and the underlying lymphoid tissue^42^. The epithelial lining of the crypts is enriched for M cells^45^ that have been shown to play an important role in sampling and transporting antigens from the crypt lumen. The base of the crypts leads to dense clusters of germinal centers that are embedded in the diffuse lymphoid tissue. Studies have shown that precursor cells from inductive lymphoid tissue constantly traffic between other mucosal sites^46,47^. Additionally, oral mucosa is highly vascularized with a high blood flow rate^48^ suggesting the tonsil vaccination can simultaneously induce potent immune responses in peripheral blood, and distal mucosal sites such as the rectal or vaginal mucosa. Numerous studies have shown that non-traumatic application of SIV on the tonsils leads to rapid infection and dissemination, whereas a single non-traumatic application of attenuated SIVmac239 on the tonsils offered partial protection against rectal challenge with SIVmac251^49–51^. Others have shown that oral immunization induced both systemic and mucosal immune responses^10,11,52–56^.

We observed a VE of 43% relative to control animals suggesting that tonsil vaccination was effective in preventing acquisition in nearly half the number of animals (Fig. 2a). We were unable to determine the exact correlates of mucosal protection from SIV acquisition, as we could not sample mucosal tissues due to the nature of weekly challenges and the potential for inducing inflammation that in turn would have driven acquisition of infection. However, a VE of 43% suggests that protective responses were likely induced in the rectal mucosa that protected from acquisition; 3/7 vaccinated animals, resisted SIV challenge till the 9th dose when all the control animals became infected. Of the 3 uninfected vaccinated animals, one animal each became positive after the 11th and 19th challenges whereas one animal remained free of SIV infection even after 22 challenges. Analysis of pre-infection immune responses in these 3 animals did not reveal any specific association with acquisition. Additional larger studies are needed to address this question in more detail. Though we could not sample mucosal sites during the study, we observed a significant increase in SIVmac239 specific IgA titres in the serum of tonsil vaccinated animals at 1-week post MVA prior to challenge (Suppl. Fig. 6). It is possible that plasmid DNA prime alone would have afforded the protection we observed in our study as we did not include a plasmid DNA only group in the study, though this is unlikely based on data from earlier studies^57–62^.

Tonsil vaccinated animals experienced a significant suppression of acute plasma viremia (~2 logs) as compared to the control animals (Fig. 2b) suggesting a role for vaccine induced anamnestic responses in acute viral control. Lower viremia was accompanied by a significant decrease in the pro-inflammatory cytokine (MCP-1, I-TAC, IL-1RA and Eotaxin) levels in the plasma (Fig. 2e) suggesting that suppression of viremia was likely associated with lower immune activation as compared to the control animals. Surprisingly, we did not detect any neutralizing activity against the challenge strain of SIVmac251 though we observed a 4 log increase in anamnestic neutralizing antibody responses against the SIVmac251.6 strain that had a significant inverse correlation with acute plasma viremia (Fig. 3c, d) suggesting that non-neutralizing activity likely contributed to the acute suppression of plasma viral loads. We did not genotype the animals for all the MHC I alleles. As such, it is difficult to rule out the role of alleles such as Mamu B*08 in the suppression of acute viremia we observed in our study. Mamu B*08 that has been associated with maintenance of lower viremia in SIV infected macaques.

Examination of V1V2 specific responses showed that tonsil vaccination induced significant levels of SIVmac251WY30 and SIVmac239CS.23 gp70 VIV2 specific antibody responses prior to infection that were significantly boosted following infection (Fig. 4a, b). A similar response was observed against the heterologous SIVsmE660 isolate suggesting that tonsil vaccination induced high levels of non-nAb with potential activity against diverse isolates of SIV (Fig. 4c, d). Induction of V1V2 specific antibody responses was associated with significant levels of ADCC activity that negatively correlated with plasma viremia (Fig. 5a, e) suggesting a role for ADCC responses in control of acute viremia. This is in line with earlier studies and the R144 trial showing an inverse correlation between ADCC and HIV infection^22,23,28–31,33,34^. Other studies have reported induction of higher V2 specific responses in macaques that correlated with protection from acquisition^25–27^. Higher ADCC responses were associated with an increase in the absolute numbers of NK cells (Fig. 5b) that expressed significantly higher levels of perforin. These perforin^+^ NK cells were Ki-67^−^ (Fig. 5d) and inversely correlated with plasma viremia (Fig. 5h) and positively correlated with ADCC titres (Fig. 5f) suggesting that non-proliferating NK cells significantly contributed to the control of acute viremia in vaccinated animals. Interestingly, Perforin^+^ NK cells that were Ki-67^+^ were found to positively correlate with plasma viremia (Fig. 5g), suggesting that these peripheral blood subsets were likely a marker of acute immune activation seen during SIV infection. Recent studies have shown that enhanced proliferation of follicular NK cells was associated with control of chronic SHIV infection^63^. Further studies are needed to clarify the role of proliferating peripheral NK subsets expressing high levels of perforin in SIV infection.

The exact reason for the higher numbers of non-proliferating NK cell subsets in vaccinated animals are not clear. We did, however, observe a significant increase in the levels of plasma IL-12 in vaccinated animals unlike the control animals (Fig. 2e) that could have played a role. Studies have shown that IL-12 plays a role in NK cell activation in healthy and HIV infected subjects^64^, whereas Wang et al.^65^ demonstrated that IL-12 along with IL-15 increased degranulation by NK cells in HIV infected subjects. Moukambi et al.^66^ on the other hand showed that SIV infected rhesus macaques displayed lower levels of IL-12 in mesenteric lymph nodes. Likewise, Gasper et al.^67^ reported that HIV infection was accompanied by lower levels of IL-12 secreting monocytes whereas their frequencies were increased in non-pathogenic SIV infection.

T cell responses play a central role in the viral control during HIV and SIV infections as numerous studies have documented. Acute viral control was accompanied by the induction of significant poly-functional SIV-env and gag specific CD8 T cell responses at 2 weeks PI as compared to control animals (Figs. 7 and 8) that were dominated by SIV specific IFNγ producing CD8 T cells. Though SIV specific CD4 T cells did not significantly differ between the control and vaccinated animals, vaccinated animals had higher levels of IFNγ responses as compared to the control animals (red arc; Figs. 7a and 8a). We could not examine T cell responses after infection in the rectal mucosa due to the nature of the weekly challenges. However, tonsil vaccinated animals had detectable T cell responses in the rectal mucosa after challenge, though these responses did not differ significantly between the two groups most likely due to the time of sampling. Interestingly, CD4 T cells in the rectal mucosa trended towards higher levels of SIV specific IL-2 and IFNγ production as compared to control animals.

Given the differences in the type of vectors, immunogens, vaccination regimen and the type of challenge stock used in previously reported studies, it is somewhat difficult to directly compare the findings reported in our study with those reported earlier. However, numerous studies have reported a significant benefit in oral vaccination against HIV in macaques. Jones et al.^10^ examined the effect of needle free immunization of the sublingual and buccal tissue using MVA-HIVgag, pol and env prime followed by a recombinant gp120 boost and reported induction of HIV specific IgG responses in vaginal, salivary and rectal secretions that was associated with delayed acquisition of SHIV-SF162P3 challenge. Delayed acquisition was found to correlate with gp120 V2 specific antibodies as in our study along with non-nAb responses. Unlike our study where one tonsil vaccinated animal was free from infection after 22 challenges, and two animals resisted infection till the 11th and 19th challenge with highly pathogenic SIVmac251, all the animals reported by Jones et al. were infected after 6 challenges with SHIV-SF162P3. In a follow up study, Sahoo et al.^55^ compared needle free oral vaccination with MVA and trimeric gp120 to intramuscular vaccination and reported similar levels of protection in both the groups with 40% of macaques being protected after 6 challenges as compared to control animals. Likewise, in a study by Curtis et al.^11^, neonatal macaques were co-administered plasmid DNA prime followed by MVA using a combination of oral and intramuscular routes with 2/6 animals delaying acquisition till the 11th and 12th challenge as compared to the control animals all of whom became infected by the 8th challenge. Delayed acquisition from SIVmac251 was associated with SIV gp120 and V1V2 specific antibody responses. Vagenas et al.^68^ reported that tonsil vaccination with inactivated AT-2 SIVmac239 particles in combination with CPG adjuvants to the tonsils partially protected macaques from SIVmac239 challenge. Likewise, spraying of the tonsils with single cycle SIV provided limited protection from oral challenge as compared to controls^69^.

Taken together, in this work we provide proof of concept that intranodal tonsil vaccination with an MVA vaccine encoding SIVmac239-env and gag complements systemic vaccination to induce potent and balanced innate and adaptive immune responses that can protect not only from acquisition from a highly pathogenic SIVmac251 infection but also rapidly control acute viremia in cases of breakthrough infections. Though the vaccine we used did not afford long-term protection most likely due to viral escape, the significant control of acute viremia suggests that tonsil vaccine induced anamnestic responses exerted a potent control on acute viremia.

## Methods

### Ethics statement

The animals were housed in accordance with the American Association for Accreditation of Laboratory Animal guidelines at Bioqual, Inc. (Kensington, MD), and all the procedures were performed according to the protocol approved by the Institutional Animal Use and Care Committee at Bioqual and accepted by the Uniformed Services University of the Health Sciences.

### Animals, vaccination, infection, and samples

Thirteen (4−7 years old) Mamu A*01^−^ male rhesus macaques of Indian origin (unvaccinated control; *n* = 6 and vaccinated; *n* = 7) that were seronegative for Vaccinia virus (determined in the laboratory of Dr. Rama Amara at Emory University), SIV, simian retrovirus (SRV), Herpes-B and simian T-cell leukemia virus (STLV) type-1 were used in this study.

Rhesus macaques in the vaccinated group were primed with 4 mg of plasmid DNA (pCDNA3) encoding SIVmac251-env and gag genes intramuscularly (IM) into the quadriceps muscle by electroporation (ECM830) at 0, 4 and 8 weeks^41,70,71^. The animals were boosted by direct intranodal injection into the tonsils using a 27” gauge needle at week 24 with 2 × 10^8^ pfu of MVA encoding SIVmac251-env and gag each.

Plasmid DNA and MVA vaccines encoding SIVmac251-env and gag genes were synthesized in Dr. Gerd Sutter’s laboratory at the Ludwig Maximilians University, Munich, Germany. Plasmid DNA was generated by ligating SIVmac251-env or SIVmac251-gag gene sequences into the vector plasmid pcDNA3.1 + (Invitrogen) under the transcriptional control of the human cytomegalovirus immediate-early promoter. Transfections of DNA preparations into 293 cells and western blot analyses were used to quality control for SIVmac251 env and gag protein expression.

The generation of recombinant MVA expressing the Green Fluorescent Protein (GFP) was described previously^72^. The MVA vector vaccines encoding SIVmac251-env and SIVmac251-gag were generated by homologous recombination as described previously^73^. Briefly, the MVA clonal isolate MVA F6^74^ served as parental virus in infection experiments. Transfection of the MVA vector plasmid pLW-73^75^ was used to direct the insertion of SIVmac251-env or SIVmac251-gag gene sequences under the transcriptional control of the synthetic vaccinia virus early/late promoter PmH5 into the intergenic site between the open reading frames MVA069R and MVA070L of the MVA genome. Recombinant MVA viruses were clonally isolated in plaque purifications in chicken embryo fibroblasts (CEF) screening for GFP expressing cell foci. Final recombinant viruses were amplified in CEF. Quality control experiments were performed using standard methodology^73^. Briefly, the genetic identity and genetic stability of the vector viruses were assessed by PCR analysis of genomic viral DNA. The replicative capacity of the recombinant viruses was tested by one-step and multiple-step growth experiments in CEF and confirmed to be very comparable to that of non-recombinant MVA. Purified stocks of MVA vector vaccines were produced by ultracentrifugation through 36% sucrose cushions followed by resuspension in 10 mM Tris buffer pH 9, titered and stored at −80 °C.

Eight weeks after the MVA boost, both the vaccinated and unvaccinated control group of animals were challenged intrarectally (IR) at weekly intervals with a repeated low dose of SIVmac251 (Ron Desrosiers 2006, Day 9 stock at 1:500 dilution; 120 TCID_50_/dose, obtained from Nancy Miller at SVEU, NIAID). The MID_50_ (50% macaque infectious dose) of virus stock required for IR transmission was determined by titration in rhesus macaques at Advanced Bioscience Laboratories (ABL, Inc. Rockville, MD) and confirmed by single genome amplification (SGA) analysis (ABL). Animals were challenged till they tested positive for plasma viral loads. Peripheral blood samples were collected prior to each challenge for determining viral loads. Plasma viral loads were determined by ABL using the NASBA method described previously^76,77^ with ≥50 SIV RNA copies/ml deemed as infection onset.

PBMC were isolated by density gradient centrifugation and cells from LN were isolated by mechanical disruption^78–83^. Rectal biopsies were collected prior to challenge but not during the challenge phase. Cells were isolated by enzymatic digestion and percoll gradient centrifugation as per procedures described previously^79,84–88^.

### Antibody assays

Serum binding antibody titer to monomeric SIVmac239-gp120 was determined at ABL Inc., by ELISA as described previously^89^. The antibody titer was defined as the reciprocal of the serum dilution at which the optical density of the test serum was two times higher than the negative-control serum that was diluted at 1:50.

Neutralizing antibody activity was measured in 96-well culture plates by using Tat-regulated luciferase (Luc) reporter gene expression to quantify reductions in virus infection in TZM-bl cells as described previously^90^. TZM-bl cells were obtained from the NIH AIDS Research and Reference Reagent Program, as contributed by John Kappes and Xiaoyun Wu. Assays were performed with replication-competent SIVmac251 Stock 8 (challenge Stock 8, 2012 Desrosiers) as well as with pseudotyped virus SIVmac251.6 as described previously^90^. Test samples were diluted over a range of 1:20 to 1:43740 or 1:30 to 1:2343750 in cell culture medium and pre-incubated with virus (~150,000 relative light unit equivalents) for 1 h at 37 °C before addition of cells, and tested in duplicate. Following a 48 h incubation, cells were lysed and Luc activity was determined using a microtiter plate luminometer and BriteLite Plus Reagent (Perkin Elmer). Neutralization titers are reported as the reciprocal sample dilution at which relative luminescence units (RLU) were reduced by 50% compared to RLU in virus control wells after subtraction of background RLU in cell control wells. Serum samples were heat-inactivated at 56 °C for 30 min prior to assay.

Plasma SIV specific antibodies to gp70-SIVsmE660-BR-CG7V V1/V2, gp70-SIVmac251-WY30 V1/V2, gp70-SIVmac239-cs.23 V1V2, gp130 SIVmac251, and gp140 SIVsmE660 were measured prior to at and 2 weeks post challenge using a modified custom HIV-1 binding antibody multiplex assay (BAMA) as described previously^91,92^. Briefly, carboxylated fluorescent beads (Luminex Corp., Austin, TX) were covalently coupled to SIV-env gp120 proteins and gp70 V1V2 scaffolds and were incubated with serially diluted (starting 1:80, 5-fold) serum samples. Antigen-specific IgG was detected using biotinylated goat anti-rhesus IgG, followed by an incubation with streptavidin PE. Antibody measurements were acquired on a Bio-Plex instrument (Bio-Rad, Hercules, CA) using 21CFR Part 11 compliant software and the readout was in MFI. Polyclonal IgG purified from a SIV-positive macaque (kindly provided by Mario Roederer, VRC), was used as a positive control. Blank beads and baseline (pre-vaccination) samples were included as negative controls in every assay. Positivity criteria were: (1) MFI > antigen-specific cutoffs (95 percentile of baseline sample binding values for each antigen), (2) MFI before non-specific binding (blank or MuLV gp70) subtraction >2*MFI of non-specific binding for the same sample, (3) MFI for post immunization samples >3*MFI for pre-immunization samples, at dilution 1:80, both before and after non-specific binding subtraction, and 4) MFI ≥ 100.

Serum IgA levels were determined prior to and at 1-week post MVA by ELISA. Briefly, 50 µl area plates were coated overnight with 2.5 µg ml of SIVmac239 gp140 foldon trimer (kind gift from Rosemarie Mason and Peter Kwong at the Vaccine Research Center, NIAID). After blocking, serially diluted serum samples were added to each well. SIVmac239 gp120 specific IgA was detected using goat anti-monkey IgA (specific against cynomolgus and rhesus macaque IgA) detection antibody conjugated to HRP (Life Diagnostics, Inc., West Chester, PA) that was cross-adsorbed to remove IgM and IgG reactivity. The plates were developed and the optical densities (O.D.) were measured at 450 nm using Softmax Pro software (Molecular Devices, LLC. San Jose, CA).

### ADCC assays

Antibody dependent cellular cytotoxicity (ADDC) assay were performed as described previously^93^. Briefly, CEM-NK^R^ cells were coated with SIVmac239-gp120 used as targets with human effector PBMC at an effector-to-target (E: T) ratio of 50:1, and mixed with serially diluted serum samples. Unstained and single-stained target cells were used as controls. Percent ADCC was determined by back-gating on the PKH-26^high^ population of target cells that were negative for the CFSE viability dye. ADCC titres were reported as reciprocal of the dilution at which the % ADCC killing was three standard deviations greater than the mean % killing of the negative control samples. The maximum % killing for each sample was determined and reported as the reciprocal serum dilution at which 50% maximum killing was observed.

### IFNγ ELISPOT assay

ELISPOT assay was performed as per manufacturer’s instructions. The 96-well Multiscreen-IP membrane plates (Millipore, Bedford, MA) were coated with 100 μl of an anti-human recombinant IFN-γ monoclonal antibody (catalog no. M-700A; Endogen, Woburn, Mass.) at a concentration of 5 μg/ml and incubated overnight at 4 °C. After washing, 200 μl of complete RPMI-10 was added to each well and incubated at 37 °C with 5% CO_2_ for 1 h to block non-specific binding. After washing, 50 μl of complete medium containing overlapping SIV-env and gag peptides were added to the appropriate wells followed by 10^5^ PBMC in 50 µl of complete RPMI-10. SEB stimulated wells were set up simultaneously as positive controls. The plates were incubated overnight at 37 °C in the presence of 5% CO_2_, and 95% humidity. Plates were washed and labeled with biotinylated anti-human recombinant IFN-γ antibody overnight at 4 °C. After washing, Streptavidin-alkaline phosphatase was added to each well. The plate was incubated for 1 h at room temperature, washed and 1-Step Nitroblue tetrazolium–5-bromo-4-chloro-3-indolylphosphate substrate was added to each well, and the spots were developed for 3 to 15 min at room temperature. After decanting the substrate, the plate was rinsed with water to stop the reaction and air-dried. The number of spots in each well was counted using the CTL Immunospot reader.

### Plasma cytokines

Plasma cytokine profiles were determined using the Monkey Cytokine Magnetic 29-Plex Panel for Luminex™ Platform (Thermofisher Scientific, Waltham, MA) at the Duke Human Vaccine Institute as per manufacturer’s instructions. The kit is designed to quantify 29 cytokines namely, EGF, Eotaxin, FGF-basic, G-CSF, GM-CSF, HGF, IFN-γ, IL-1β, IL-1RA, IL-2, IL-4, IL-5, IL-6, IL-8, IL-10, IL-12, IL-15, IL-17, IP-10, I-TAC, MCP-1, MDC, MIF, MIG, MIP-1α, MIP-1β, RANTES, TNF-α, and VEGF. This kit has been successfully used to identify cytokines in rhesus macaque plasma in earlier studies^94,95^. Briefly, plasma samples were diluted 1: 2 in assay diluent as per manufacturer’s instructions and used to quantify each cytokine. The plates were analyzed using Luminex xMAP technology on a Bio-plex 200 system (Biorad), and concentrations were determined using Bioplex manager software 6.1.

### Antibodies and flow cytometry

For phenotypic analysis, cells were labeled simultaneously with a panel of anti-CD3-Cy-7APC (clone SP34-2; 1:300 dilution), CD4-APC (clone RPA-T4; 1:300 dilution), CD8-Alexa700 (clone RPA-T8; 1:300 dilution), CD95-FITC (clone DX2; 1:300 dilution) and CD28-Cy-5PE (clone CD28.2; 1:300 dilution) antibodies (BD Biosciences). Memory CD4 T cells were discriminated based on the expression of CD28 and CD95 as described previously^96,97^. To identify NK cells, PBMC were surface labeled with a panel of anti-CD3 (1:300 dilution), CD20 (clone 2H7; 1:300 dilution), CD14 (clone M5E2; 1:300 dilution), NKG2A (clone Z199; 1:300 dilution), and KIR2D (clone NKVFS1; 1:300 dilution) antibodies. After the cells were fixed and permeabilized, they were labeled with anti-perforin (clone deltaG9; 1:100 dilution) and Ki-67 (clone B56; 1:300 dilution) antibodies. Labeled cells were fixed with 0.5% paraformaldehyde and analyzed using an LSR II flow cytometer (BD Biosciences). The gating strategy is shown in Supplementary Fig. 5. All the antibodies were titrated using rhesus macaque PBMC.

To determine SIV-env and gag specific CD4 and CD8 T cell responses, cells were stimulated with overlapping peptides as described previously^41,81,98^. Control cultures were set up for each sample without SIV peptides. After stimulation, cells were labeled with cell surface markers (anti-CD3, CD4, CD8, CD28 and CD95 at dilutions as above) and Vivid to discriminate live and dead cells^99^. The cells were fixed (Fix/Perm kit; BD Biosciences), and after permeabilization were labeled with anti-IL-2-PE (clone MQ1-17H12; 1:300 dilution), IFN-γ-FITC (clone B27; 1:300 dilution), and TNF-α-Cy7PE (clone Mab11; 1:50 dilution). Labeled cells were fixed with 0.5% paraformaldehyde and analyzed using an LSR II flow cytometer (BD Biosciences).

### Data analysis

Flow cytometric data were analyzed using FlowJo version 9.6 (Tree Star, Inc., Ashland, OR). Statistical analysis was performed using GraphPad Prism Version 9.0 (GraphPad Prism Software, Inc. San Diego, CA) and SPICE software^100^. Rate of SIV acquisition after each challenge was determined using Kaplan–Meier plots and vaccine efficacy (VE) after the 9th IR challenge was determined using the formula VE = 1 – [(infection rate among vaccinated animals)/(infection rate among control animals)] × 100 and followed by Fisher’s exact test as described by Klasse et al.^101^. Statistical difference between groups was determined using Mann–Whitney *U* test and a Spearman’s rank test was used to determine correlations. A *P* < 0.05 was considered significant. Error bars represent standard error.

### Reporting summary

Further information on research design is available in the Nature Portfolio Reporting Summary linked to this article.

## Supplementary information

Supplementary Information

Reporting summary

## Data Availability

All data in the manuscript are reported in the figures and are available upon request.

## References

[1] Baral S, Sifakis F, Cleghorn F, Beyrer C (2007). Elevated risk for HIV infection among men who have sex with men in low- and middle-income countries 2000-2006: a systematic review. PLoS Med..

[2] Harmon TM (2016). Exploring the potential health impact and cost-effectiveness of AIDS vaccine within a comprehensive HIV/AIDS response in low- and middle-income countries. PLoS ONE.

[3] Stover J (2014). How can we get close to zero? The potential contribution of biomedical prevention and the investment framework towards an effective response to HIV. PLoS ONE.

[4] Denning PH, Campsmith ML (2005). Unprotected anal intercourse among HIV-positive men who have a steady male sex partner with negative or unknown HIV serostatus. Am. J. Public Health.

[5] Brandtzaeg P, Farstad IN, Haraldsen G (1999). Regional specialization in the mucosal immune system: primed cells do not always home along the same track. Immunol. Today.

[6] Cesta MF (2006). Normal structure, function, and histology of mucosa-associated lymphoid tissue. Toxicol. Pathol..

[7] Kunisawa J, Fukuyama S, Kiyono H (2005). Mucosa-associated lymphoid tissues in the aerodigestive tract: their shared and divergent traits and their importance to the orchestration of the mucosal immune system. Curr. Mol. Med..

[8] Barnett SW (2008). Protection of macaques against vaginal SHIV challenge by systemic or mucosal and systemic vaccinations with HIV-envelope. AIDS.

[9] Zhou Q (2007). Comparative evaluation of oral and intranasal priming with replication-competent adenovirus 5 host range mutant (Ad5hr)-simian immunodeficiency virus (SIV) recombinant vaccines on immunogenicity and protective efficacy against SIVmac251. Vaccine.

[10] Jones AT (2019). HIV-1 vaccination by needle-free oral injection induces strong mucosal immunity and protects against SHIV challenge. Nat. Commun..

[11] Curtis AD (2019). Oral coadministration of an intramuscular DNA/modified vaccinia Ankara vaccine for simian immunodeficiency virus is associated with better control of infection in orally exposed infant macaques. AIDS Res. Hum. Retroviruses.

[12] Manrique M (2014). Resistance to infection, early and persistent suppression of simian immunodeficiency virus SIVmac251 viremia, and significant reduction of tissue viral burden after mucosal vaccination in female rhesus macaques. J. Virol..

[13] Aarntzen EH (2012). Vaccination with mRNA-electroporated dendritic cells induces robust tumor antigen-specific CD4+ and CD8+ T cells responses in stage III and IV melanoma patients. Clin. Cancer Res..

[14] Barth RJ (2010). A randomized trial of ex vivo CD40L activation of a dendritic cell vaccine in colorectal cancer patients: tumor-specific immune responses are associated with improved survival. Clin. Cancer Res..

[15] Bol KF (2015). Intranodal vaccination with mRNA-optimized dendritic cells in metastatic melanoma patients. Oncoimmunology.

[16] Schwaab T (2009). Clinical and immunologic effects of intranodal autologous tumor lysate-dendritic cell vaccine with Aldesleukin (Interleukin 2) and IFN-{alpha}2a therapy in metastatic renal cell carcinoma patients. Clin. Cancer Res..

[17] Lambert LA (2001). Intranodal immunization with tumor lysate-pulsed dendritic cells enhances protective antitumor immunity. Cancer Res..

[18] Bedrosian I (2003). Intranodal administration of peptide-pulsed mature dendritic cell vaccines results in superior CD8+ T-cell function in melanoma patients. J. Clin. Oncol..

[19] Fouda GG (2017). Systemic administration of an HIV-1 broadly neutralizing dimeric IgA yields mucosal secretory IgA and virus neutralization. Mucosal. Immunol..

[20] Mascola JR (2000). Protection of macaques against vaginal transmission of a pathogenic HIV-1/SIV chimeric virus by passive infusion of neutralizing antibodies. Nat. Med..

[21] Shingai M (2014). Passive transfer of modest titers of potent and broadly neutralizing anti-HIV monoclonal antibodies block SHIV infection in macaques. J. Exp. Med..

[22] Haynes BF (2012). Immune-correlates analysis of an HIV-1 vaccine efficacy trial. N. Engl. J. Med..

[23] Zolla-Pazner S (2013). Analysis of V2 antibody responses induced in vaccinees in the ALVAC/AIDSVAX HIV-1 vaccine efficacy trial. PLoS ONE.

[24] Zolla-Pazner S, Alvarez R, Kong XP, Weiss S (2019). Vaccine-induced V1V2-specific antibodies control and or protect against infection with HIV, SIV and SHIV. Curr. Opin. HIV AIDS.

[25] Schifanella L (2019). ALVAC-HIV B/C candidate HIV vaccine efficacy dependent on neutralization profile of challenge virus and adjuvant dose and type. PLoS Pathog..

[26] Vaccari M (2018). HIV vaccine candidate activation of hypoxia and the inflammasome in CD14(+) monocytes is associated with a decreased risk of SIV(mac251) acquisition. Nat. Med..

[27] Vaccari M (2016). Adjuvant-dependent innate and adaptive immune signatures of risk of SIVmac251 acquisition. Nat. Med..

[28] Hessell AJ (2019). Multimeric epitope-scaffold HIV vaccines target V1V2 and differentially tune polyfunctional antibody responses. Cell Rep..

[29] Jones, A. T. et al. A Trimeric HIV-1 envelope gp120 immunogen induces potent and broad anti-V1V2 loop antibodies against HIV-1 in rabbits and rhesus macaques. *J. Virol.* **92**, 10.1128/JVI.01796-17 (2018). 10.1128/JVI.01796-17 PMC5809733 29237847

[30] Malherbe, D. C. et al. Combination adenovirus and protein vaccines prevent infection or reduce viral burden after heterologous clade C simian-human immunodeficiency virus mucosal challenge. *J. Virol.* **92**, 10.1128/JVI.01092-17 (2018). 10.1128/JVI.01092-17 PMC5752948 29093095

[31] Weiss S (2022). Differential V2-directed antibody responses in non-human primates infected with SHIVs or immunized with diverse HIV vaccines. Nat. Commun..

[32] Singh, S. et al. Control of heterologous simian immunodeficiency virus SIVsmE660 infection by DNA and protein coimmunization regimens combined with different toll-like-receptor-4-based adjuvants in macaques. *J. Virol.* **92**, 10.1128/JVI.00281-18 (2018). 10.1128/JVI.00281-18 PMC6052320 29793957

[33] Rerks-Ngarm S (2009). Vaccination with ALVAC and AIDSVAX to prevent HIV-1 infection in Thailand. N. Engl. J. Med..

[34] Rolland M (2012). Increased HIV-1 vaccine efficacy against viruses with genetic signatures in Env V2. Nature.

[35] Jin X (1999). Dramatic rise in plasma viremia after CD8(+) T cell depletion in simian immunodeficiency virus-infected macaques. J. Exp. Med..

[36] Kostense S (2002). Functional restoration of human immunodeficiency virus and Epstein-Barr virus-specific CD8(+) T cells during highly active antiretroviral therapy is associated with an increase in CD4(+) T cells. Eur. J. Immunol..

[37] Koup RA (1994). Temporal association of cellular immune responses with the initial control of viremia in primary human immunodeficiency virus type 1 syndrome. J. Virol..

[38] Lifson JD (2001). Role of CD8(+) lymphocytes in control of simian immunodeficiency virus infection and resistance to rechallenge after transient early antiretroviral treatment. J. Virol..

[39] Matano T (1998). Administration of an anti-CD8 monoclonal antibody interferes with the clearance of chimeric simian/human immunodeficiency virus during primary infections of rhesus macaques. J. Virol..

[40] Schmitz JE (1999). Control of viremia in simian immunodeficiency virus infection by CD8+ lymphocytes. Science.

[41] Mattapallil JJ (2006). Vaccination preserves CD4 memory T cells during acute simian immunodeficiency virus challenge. J. Exp. Med..

[42] Nair PN, Rossinsky K (1984). Crypt architecture of tonsilla lingualis in the monkey, *Macaca fascicularis*. A correlated light- and scanning electron-microscopic study. Cell Tissue Res..

[43] Gleason, E. B. *A Manual of Diseases of the Nose, Throat and Ear*, 4th edn. (W. B. Saunders Company, 1918).

[44] Gray, H. *Anatomy: Descriptive and Applied*, 18th edn. (Lea and Febiger, 1910).

[45] Perry M, Whyte A (1998). Immunology of the tonsils. Immunol. Today.

[46] McGhee JR, Mestecky J, Elson CO, Kiyono H (1989). Regulation of IgA synthesis and immune response by T cells and interleukins. J. Clin. Immunol..

[47] Ogra PL, Faden H, Welliver RC (2001). Vaccination strategies for mucosal immune responses. Clin. Microbiol. Rev..

[48] Lu FX, Jacobson RS (2007). Oral mucosal immunity and HIV/SIV infection. J. Dent. Res..

[49] Stahl-Hennig C (2007). A single vaccination with attenuated SIVmac 239 via the tonsillar route confers partial protection against challenge with SIVmac 251 at a distant mucosal site, the rectum. Front. Biosci.

[50] Stahl-Hennig C (1999). Rapid infection of oral mucosal-associated lymphoid tissue with simian immunodeficiency virus. Science.

[51] Tenner-Racz K (2004). Early protection against pathogenic virus infection at a mucosal challenge site after vaccination with attenuated simian immunodeficiency virus. Proc. Natl Acad. Sci. USA.

[52] Desvignes C (1998). The murine buccal mucosa is an inductive site for priming class I-restricted CD8+ effector T cells in vivo. Clin. Exp. Immunol..

[53] Etchart N, Buckland R, Liu MA, Wild TF, Kaiserlian D (1997). Class I-restricted CTL induction by mucosal immunization with naked DNA encoding measles virus haemagglutinin. J. Gen. Virol..

[54] Makitalo B (2004). Enhanced cellular immunity and systemic control of SHIV infection by combined parenteral and mucosal administration of a DNA prime MVA boost vaccine regimen. J. Gen. Virol..

[55] Sahoo A (2022). A clade C HIV-1 vaccine protects against heterologous SHIV infection by modulating IgG glycosylation and T helper response in macaques. Sci. Immunol..

[56] Velarde de la Cruz E (2021). Oral vaccination approaches for Anti-SHIV immunity. Front. Immunol..

[57] Graham BS (2006). Phase 1 safety and immunogenicity evaluation of a multiclade HIV-1 DNA candidate vaccine. J. Infect. Dis..

[58] MacGregor RR (1998). First human trial of a DNA-based vaccine for treatment of human immunodeficiency virus type 1 infection: safety and host response. J. Infect. Dis..

[59] Robinson HL, Hunt LA, Webster RG (1993). Protection against a lethal influenza virus challenge by immunization with a haemagglutinin-expressing plasmid DNA. Vaccine.

[60] Roy MJ (2000). Induction of antigen-specific CD8+ T cells, T helper cells, and protective levels of antibody in humans by particle-mediated administration of a hepatitis B virus DNA vaccine. Vaccine.

[61] Ulmer JB (1993). Heterologous protection against influenza by injection of DNA encoding a viral protein. Science.

[62] Xiang ZQ (1994). Vaccination with a plasmid vector carrying the rabies virus glycoprotein gene induces protective immunity against rabies virus. Virology.

[63] Rahman, S. A. et al. Lymph node CXCR5+ NK cells associate with control of chronic SHIV infection. *JCI Insight* **7**, 10.1172/jci.insight.155601 (2022). 10.1172/jci.insight.155601 PMC9089783 35271506

[64] Ferlazzo G (2004). Distinct roles of IL-12 and IL-15 in human natural killer cell activation by dendritic cells from secondary lymphoid organs. Proc. Natl Acad. Sci. USA.

[65] Wang Y (2020). HIV-1-induced cytokines deplete homeostatic innate lymphoid cells and expand TCF7-dependent memory NK cells. Nat. Immunol..

[66] Moukambi F (2019). Mucosal T follicular helper cells in SIV-infected rhesus macaques: contributing role of IL-27. Mucosal. Immunol..

[67] Gasper MA (2016). Nonpathogenic SIV and pathogenic HIV infections associate with disparate innate cytokine signatures in response to *Mycobacterium bovis* BCG. PLoS ONE.

[68] Vagenas P (2009). Tonsillar application of AT-2 SIV affords partial protection against rectal challenge with SIVmac239. J. Acquir. Immune Defic. Syndr..

[69] Stahl-Hennig C (2007). Atraumatic oral spray immunization with replication-deficient viral vector vaccines. J. Virol..

[70] Letvin NL (2004). Heterologous envelope immunogens contribute to AIDS vaccine protection in rhesus monkeys. J. Virol..

[71] Mascola JR (2005). Neutralizing antibodies elicited by immunization of monkeys with DNA plasmids and recombinant adenoviral vectors expressing human immunodeficiency virus type 1 proteins. J. Virol..

[72] Staib C (2000). Transient host range selection for genetic engineering of modified vaccinia virus Ankara. Biotechniques.

[73] Kremer M (2012). Easy and efficient protocols for working with recombinant vaccinia virus MVA. Methods Mol. Biol..

[74] Meyer H, Sutter G, Mayr A (1991). Mapping of deletions in the genome of the highly attenuated vaccinia virus MVA and their influence on virulence. J. Gen. Virol..

[75] Wyatt LS (2009). Elucidating and minimizing the loss by recombinant vaccinia virus of human immunodeficiency virus gene expression resulting from spontaneous mutations and positive selection. J. Virol..

[76] Lee EM (2010). Molecular methods for evaluation of virological status of nonhuman primates challenged with simian immunodeficiency or simian-human immunodeficiency viruses. J. Virol. Methods.

[77] Romano JW (2000). Quantitative evaluation of simian immunodeficiency virus infection using NASBA technology. J. Virol. Methods.

[78] Kader M, Bixler S, Piatak M, Lifson J, Mattapallil JJ (2009). Anti-retroviral therapy fails to restore the severe Th-17: Tc-17 imbalance observed in peripheral blood during simian immunodeficiency virus infection. J. Med. Primatol..

[79] Kader M (2008). Antiretroviral therapy prior to acute viral replication preserves CD4 T cells in the periphery but not in rectal mucosa during acute simian immunodeficiency virus infection. J. Virol..

[80] Kuwata T (2006). Infectious molecular clones from a simian immunodeficiency virus-infected rapid-progressor (RP) macaque: evidence of differential selection of RP-specific envelope mutations in vitro and in vivo. J. Virol..

[81] Mattapallil JJ, Hill B, Douek DC, Roederer M (2006). Systemic vaccination prevents the total destruction of mucosal CD4 T cells during acute SIV challenge. J. Med. Primatol..

[82] Mattapallil JJ, Reay E, Dandekar S (2000). An early expansion of CD8alphabeta T cells, but depletion of resident CD8alphaalpha T cells, occurs in the intestinal epithelium during primary simian immunodeficiency virus infection. Aids.

[83] Moore AC, Bixler SL, Lewis MG, Verthelyi D, Mattapallil JJ (2012). Mucosal and peripheral Lin- HLA-DR+ CD11c/123- CD13+ CD14- mononuclear cells are preferentially infected during acute simian immunodeficiency virus infection. J. Virol..

[84] Bixler, S. L., Sandler, N. G., Douek, D. C. & Mattapallil, J. J. Suppressed Th17 levels correlate with elevated PIAS3, SHP2, and SOCS3 expression in CD4 T cells during acute simian immunodeficiency virus infection. *J. Virol*. 10.1128/JVI.00600-13 (2013). 10.1128/JVI.00600-13 PMC3676085 23596301

[85] Eberly MD (2009). Increased IL-15 production is associated with higher susceptibility of memory CD4 T cells to simian immunodeficiency virus during acute infection. J. Immunol..

[86] George J (2011). Early short-term antiretroviral therapy is associated with a reduced prevalence of CD8(+)FoxP3(+) T cells in simian immunodeficiency virus-infected controller rhesus macaques. AIDS Res. Hum. Retroviruses.

[87] Kader M (2009). Alpha4(+)beta7(hi)CD4(+) memory T cells harbor most Th-17 cells and are preferentially infected during acute SIV infection. Mucosal. Immunol..

[88] Onabajo OO, George J, Lewis MG, Mattapallil JJ (2013). Rhesus macaque lymph node PD-1(hi)CD4(+) T cells express high levels of CXCR5 and IL-21 and display a CCR7(lo)ICOS(+)Bcl6(+) T-follicular helper (Tfh) cell phenotype. PLoS ONE.

[89] Demberg T (2012). Dynamics of memory B-cell populations in blood, lymph nodes, and bone marrow during antiretroviral therapy and envelope boosting in simian immunodeficiency virus SIVmac251-infected rhesus macaques. J. Virol..

[90] Montefiori DC, Measuring HIV (2009). neutralization in a luciferase reporter gene assay. Methods Mol. Biol..

[91] Shen X (2020). HIV-1 vaccine sequences impact V1V2 antibody responses: a comparison of two poxvirus prime gp120 boost vaccine regimens. Sci. Rep..

[92] Tomaras GD (2011). Polyclonal B cell responses to conserved neutralization epitopes in a subset of HIV-1-infected individuals. J. Virol..

[93] Gomez-Roman VR (2006). A simplified method for the rapid fluorometric assessment of antibody-dependent cell-mediated cytotoxicity. J. Immunol. Methods.

[94] George J (2017). Prior exposure to Zika virus significantly enhances peak dengue-2 viremia in rhesus macaques. Sci. Rep..

[95] Valiant WG (2019). Simultaneous coinfection of macaques with Zika and dengue viruses does not enhance acute plasma viremia but leads to activation of monocyte subsets and biphasic release of pro-inflammatory cytokines. Sci. Rep..

[96] Mattapallil JJ (2005). Massive infection and loss of memory CD4+ T cells in multiple tissues during acute SIV infection. Nature.

[97] Pitcher CJ (2002). Development and homeostasis of T cell memory in rhesus macaque. J. Immunol..

[98] Maecker HT (2001). Use of overlapping peptide mixtures as antigens for cytokine flow cytometry. J. Immunol. Methods.

[99] Perfetto SP (2010). Amine-reactive dyes for dead cell discrimination in fixed samples. Curr. Protoc. Cytom..

[100] Roederer M, Nozzi JL, Nason MC (2011). SPICE: exploration and analysis of post-cytometric complex multivariate datasets. Cytometry A.

[101] Klasse PJ, Moore JP (2022). Reappraising the value of HIV-1 vaccine correlates of protection analyses. J. Virol..

