# Supplementary material for: Direct intranodal tonsil vaccination with modified vaccinia Ankara vaccine protects macaques from highly pathogenic SIVmac251
Source: Nat Commun. 2023 Mar 7;14:1264. doi: 10.1038/s41467-023-36907-0 (PMC9990026; doi:10.1038/s41467-023-36907-0)
Supplement: Supplementary file 1 — Supplementary Information [file 41467_2023_36907_MOESM1_ESM.pdf]

SUPPLEMENTARY INFORMATION

Direct Intranodal Delivery of SIV-env/gag MVA Vaccine to the Tonsils Protects Rhesus Macaques from Repeated Low Dose Intrarectal Challenge with Highly Pathogenic SIVmac251

Jeffy G. Mattathil[1], Asisa Volz[2], Olusegun O. Onabajo[1], Sean Maynard[1], Sandra L. Bixler[1], Xiaoying X. Shen[3], Diego Vargas-Inchaustegui[4], Marjorie Robert-Guroff[4], Celia Lebranche[3], Georgia Tomaras[3], David Montefiori[3], Gerd Sutter[5] & Joseph J. Mattapallil[6*]

[1]Henry M. Jackson Foundation for Military Medicine, Bethesda, MD, USA; [2]Institute of Virology, University of Veterinary Medicine Hannover, Hanover, Germany; [3]Duke University School of Medicine, Durham, NC, USA; [4]National Cancer Institute, Bethesda, MD, USA; [5]Division of Virology, Department of Veterinary Sciences, LMU, Munich, Germany; [6]Uniformed Services University, Bethesda, MD, USA

Supplementary Figure 1. Gating strategy used to identify MVA-GFP+ tonsil cells.

Cells isolated from the tonsils were infected with MVA-GFP and labeled with CD45 and analyzed by flow cytometry.

Supplementary Figure 2. Plasma viral loads in control and tonsil MVA vaccinated animals.

Supplementary Figure 3. Gating strategy used to discriminate NK cell subsets.

PBMC were surface labeled with a panel of anti-CD3, CD20, CD14, NKG2A and KIR2D antibodies. After the cells were fixed and permeabilized, they were labeled with anti-perforin and Ki-67 antibodies. Labeled cells were fixed with 0.5% paraformaldehyde and analyzed using an LSR II flow cytometer (BD Biosciences). Frequencies of NK cell subsets differentially expressing Ki67, KIR2D and Perforin within each CD3-CD20-CD14-NKG2A+ NK cell subset were determined by Boolean gating using Flowjo and formatted for presentation with Spice and Pestle software.

Supplementary Figure 4. Tonsil vaccination induced detectable levels of SIVmac239-env specific T cell responses in rectal mucosa. Qualitative analysis of SIVmac239-env specific IFNγ, IL-2, and TNFα expression in rectal mucosal a) CD4 and b) CD8 T cells of control (n = 6) and tonsil vaccinated (n = 6) animals at 4 weeks post infection. Frequency of c) $CD3^+CD4^+$ T cells and d) $CD3^+CD8^+$ T cells in rectal mucosa of control (n = 6) and tonsil vaccinated (n = 6) animals that express IFNγ, IL-2, and TNFα at 4 weeks post infection. Statistical analysis was performed using two-sided Wilcoxon Rank Sum Test in SPICE software and a $p < 0.05$ (*) was considered significant. * indicate $p < 0.05$. Error bars represent standard error.

Supplementary Figure 5. Tonsil vaccination induced detectable levels of SIVmac239-gag specific T cell responses in rectal mucosa. Qualitative analysis of SIVmac239-gag specific IFNγ, IL-2, and TNFα expression in rectal mucosal a) CD4 and b) CD8 T cells of control (n = 6) and tonsil vaccinated (n = 6) animals at 4 weeks post infection. Frequency of c) $CD3^+CD4^+$ T cells and d) $CD3^+CD8^+$ T cells in rectal mucosa of control (n = 6) and tonsil vaccinated (n = 6) animals that express IFNγ, IL-2, and TNFα at 4 weeks post infection. Statistical analysis was performed using two-sided Wilcoxon Rank Sum Test in SPICE software and a $p < 0.05$ (*) was considered significant. * indicate $p < 0.05$. Error bars represent standard error.

Supplementary Figure 6. Tonsil vaccination induced detectable levels of SIVmac239-gp140 specific IgA in the serum. SIV specific serum IgA were determined in control (n = 6) and tonsil vaccinated (n = 6) animals prior to and at 1-week post-MVA by ELISA ($p = 0.0011$). Plates were coated with SIVmac239 gp140 foldon trimer (kind gift from Rosemarie Mason and Peter Kwong at the Vaccine Research Center, NIAID)

and serum IgA levels were detected using macaque specific IgA detection antibody conjugated to HRP. Box plots show minima, 25% percentile, median, 75 percentile and maxima. Statistical difference between groups was determined using One-tailed *Mann Whitney U* test and a $p < 0.05$ was considered significant. * indicate $p < 0.05$. Error bars represent standard error.

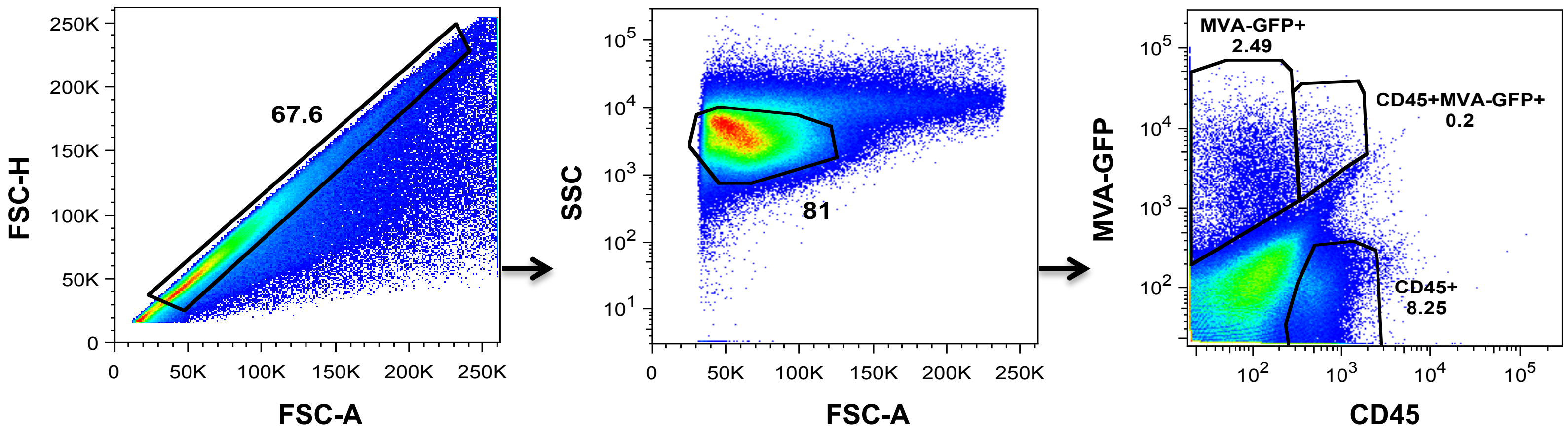

George et al Suppl. Figure 1

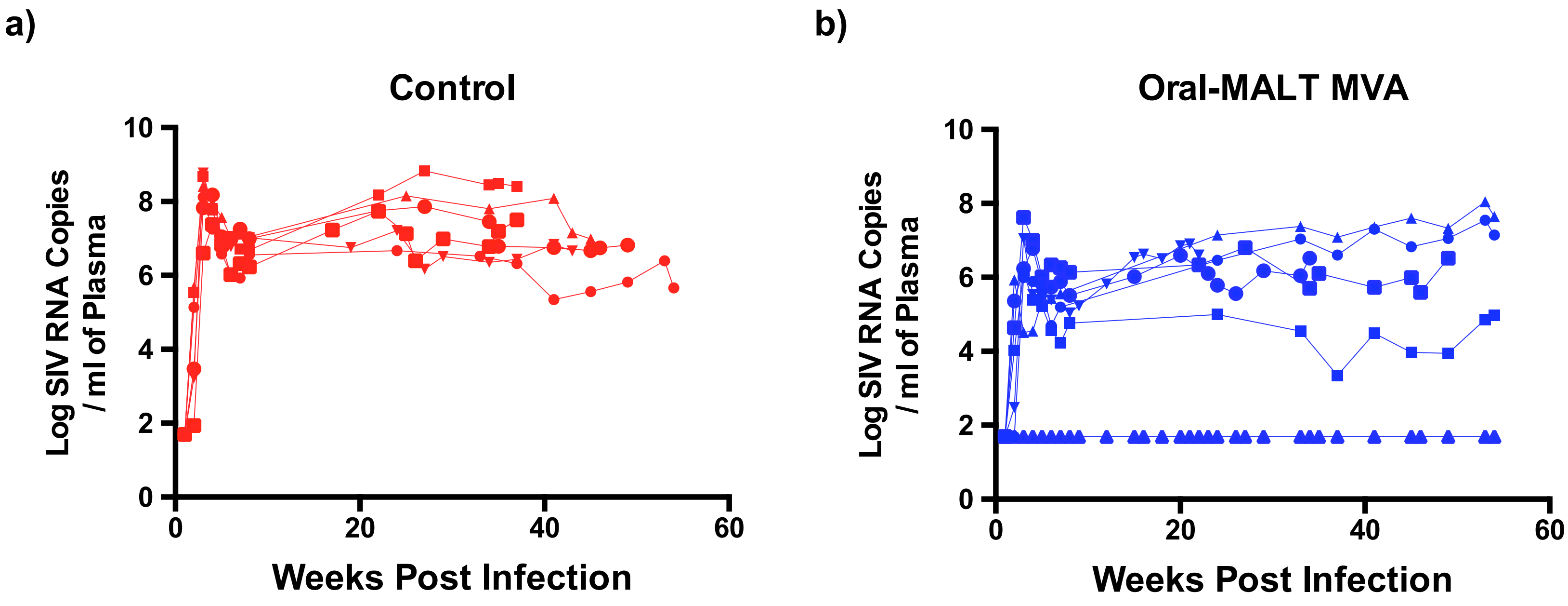

George et al Suppl. Figure 2

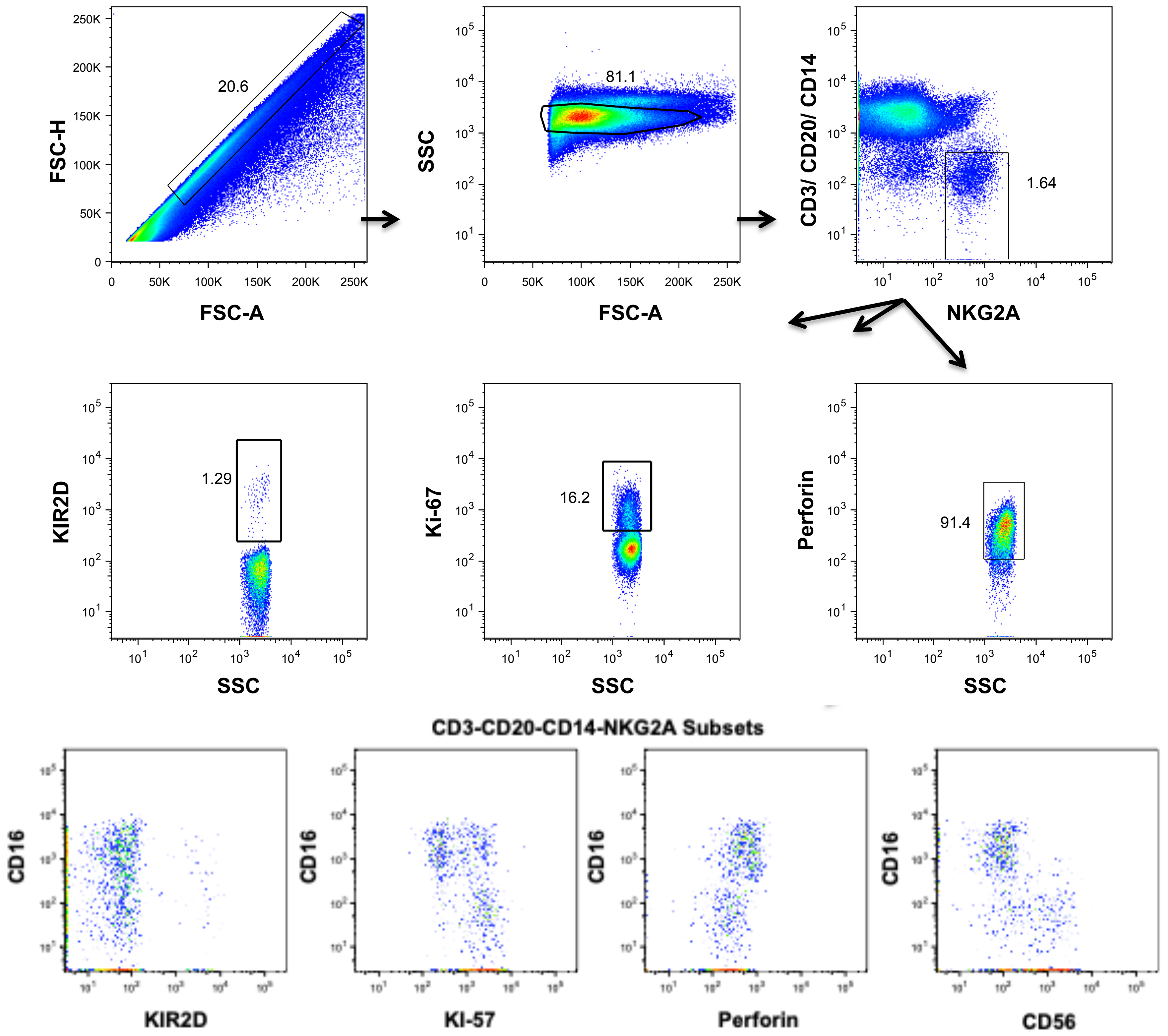

George et al Suppl. Figure 3

SIV-*env* Specific T Cell Responses in Rectal Mucosa (4 Weeks Post-infection)

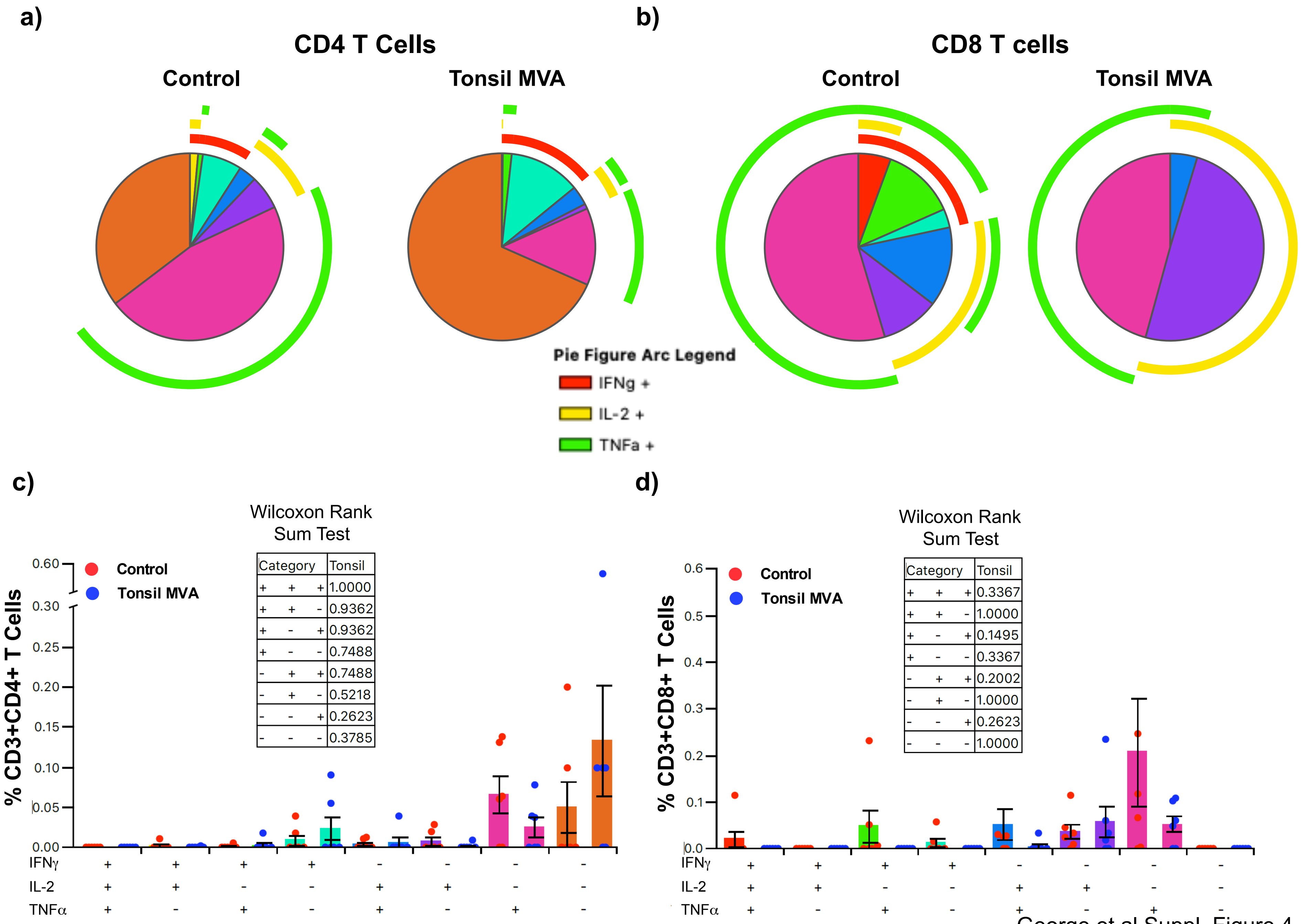

c) Wilcoxon Rank Sum Test

Category			Tonsil
+	+	+	1.0000
+	+	-	0.9362
+	-	+	0.9362
+	-	-	0.7488
-	+	+	0.7488
-	+	-	0.5218
-	-	+	0.2623
-	-	-	0.3785

d) Wilcoxon Rank Sum Test

Category			Tonsil
+	+	+	0.3367
+	+	-	1.0000
+	-	+	0.1495
+	-	-	0.3367
-	+	+	0.2002
-	+	-	1.0000
-	-	+	0.2623
-	-	-	1.0000

George et al Suppl. Figure 4

SIV-*gag* Specific T Cell Responses in Rectal Mucosa (4 Weeks Post-infection)

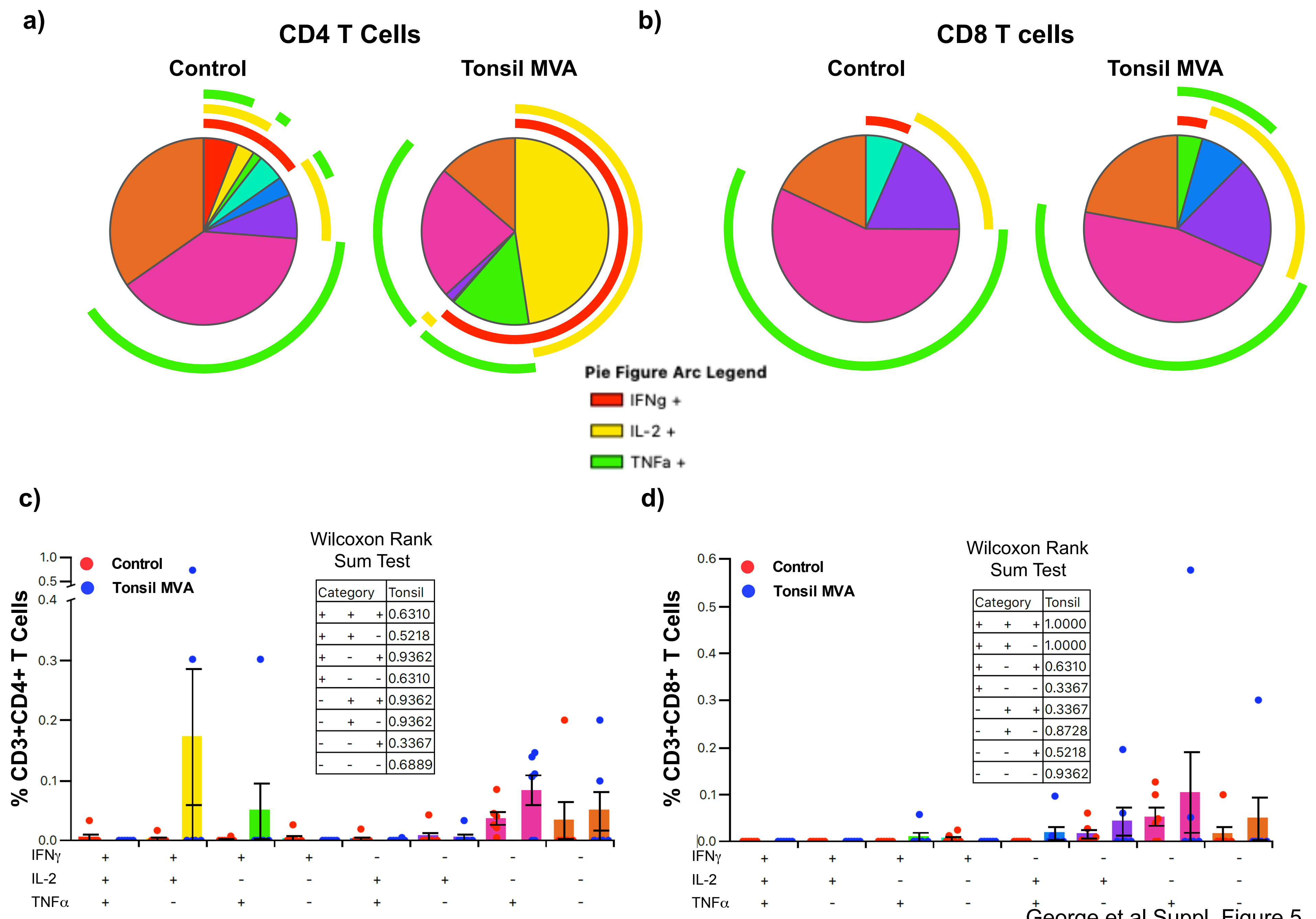

c) Wilcoxon Rank Sum Test

Category			Tonsil
+	+	+	0.6310
+	+	-	0.5218
+	-	+	0.9362
+	-	-	0.6310
-	+	+	0.9362
-	+	-	0.9362
-	-	+	0.3367
-	-	-	0.6889

d) Wilcoxon Rank Sum Test

Category			Tonsil
+	+	+	1.0000
+	+	-	1.0000
+	-	+	0.6310
+	-	-	0.3367
-	+	+	0.3367
-	+	-	0.8728
-	-	+	0.5218
-	-	-	0.9362

George et al Suppl. Figure 5

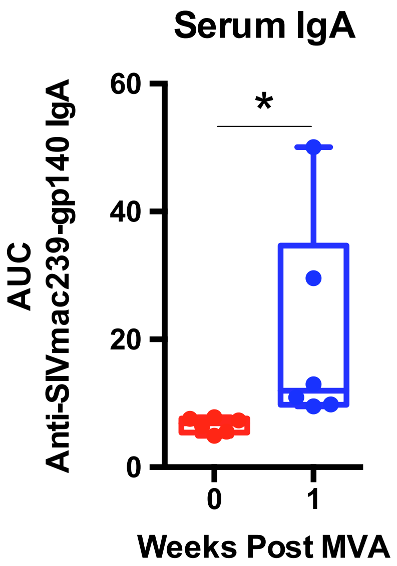

George et al Suppl. Figure 6
