# Supplementary material for: Direct intranodal tonsil vaccination with modified vaccinia Ankara vaccine protects macaques from highly pathogenic SIVmac251
Source: Nat Commun. 2023 Mar 7;14:1264. doi: 10.1038/s41467-023-36907-0 (PMC9990026; doi:10.1038/s41467-023-36907-0)
Supplement: Supplementary file 2 — Reporting summary [file 41467_2023_36907_MOESM2_ESM.pdf]

nature portfolio

Corresponding author(s): Joseph Mattapallil

Last updated by author(s): Feb 13, 2023

Reporting Summary

Nature Portfolio wishes to improve the reproducibility of the work that we publish. This form provides structure for consistency and transparency in reporting. For further information on Nature Portfolio policies, see our Editorial Policies and the Editorial Policy Checklist.

Please do not complete any field with "not applicable" or n/a. Refer to the help text for what text to use if an item is not relevant to your study.
For final submission: please carefully check your responses for accuracy; you will not be able to make changes later.

Statistics

For all statistical analyses, confirm that the following items are present in the figure legend, table legend, main text, or Methods section.

n/a	Confirmed	
☐	☒	The exact sample size (*n*) for each experimental group/condition, given as a discrete number and unit of measurement
☐	☒	A statement on whether measurements were taken from distinct samples or whether the same sample was measured repeatedly
☐	☒	The statistical test(s) used AND whether they are one- or two-sided<br>*Only common tests should be described solely by name; describe more complex techniques in the Methods section.*
☒	☐	A description of all covariates tested
☐	☒	A description of any assumptions or corrections, such as tests of normality and adjustment for multiple comparisons
☐	☒	A full description of the statistical parameters including central tendency (e.g. means) or other basic estimates (e.g. regression coefficient) AND variation (e.g. standard deviation) or associated estimates of uncertainty (e.g. confidence intervals)
☐	☒	For null hypothesis testing, the test statistic (e.g. *F*, *t*, *r*) with confidence intervals, effect sizes, degrees of freedom and *P* value noted<br>*Give P values as exact values whenever suitable.*
☒	☐	For Bayesian analysis, information on the choice of priors and Markov chain Monte Carlo settings
☒	☐	For hierarchical and complex designs, identification of the appropriate level for tests and full reporting of outcomes
☒	☐	Estimates of effect sizes (e.g. Cohen's *d*, Pearson's *r*), indicating how they were calculated

Our web collection on statistics for biologists contains articles on many of the points above.

Software and code

Policy information about availability of computer code

Data collection	No software was used to collect samples
Data analysis	GraphPad Prism 9.0, Softmax Pro 4.8

For manuscripts utilizing custom algorithms or software that are central to the research but not yet described in published literature, software must be made available to editors and reviewers. We strongly encourage code deposition in a community repository (e.g. GitHub). See the Nature Portfolio guidelines for submitting code & software for further information.

Data

Policy information about availability of data

All manuscripts must include a data availability statement. This statement should provide the following information, where applicable:

- Accession codes, unique identifiers, or web links for publicly available datasets
- A description of any restrictions on data availability
- For clinical datasets or third party data, please ensure that the statement adheres to our policy

All relevant data associated with this manuscript are in the figures and supplementary information, and additional data are available upon request.

Human research participants

Policy information about studies involving human research participants and Sex and Gender in Research.

Reporting on sex and gender	Not applicable
Population characteristics	Not applicable
Recruitment	Not applicable
Ethics oversight	Not applicable

Note that full information on the approval of the study protocol must also be provided in the manuscript.

Field-specific reporting

Please select the one below that is the best fit for your research. If you are not sure, read the appropriate sections before making your selection.

☒ Life sciences ☐ Behavioural & social sciences ☐ Ecological, evolutionary & environmental sciences

Life sciences study design

All studies must disclose on these points even when the disclosure is negative.

Sample size	Sample sizes were determined based on previously published studies. The sample sizes used was sufficient to achieve statistical differences between the vaccinated and control groups. The experiments involved samples collected longitudinally from rhesus macaques. As such the data represents biological replicates. The animal study was only performed once.
Data exclusions	No data exclusions.
Replication	All replications were successful. The experiments involved biological replicates from each group that were compared. The animal study was only performed once.
Randomization	No randomization tool was used for this experimental study. Covariates were not considered given the pilot nature of the study and the sample sizes.
Blinding	Samples were randomized and serially labeled prior to use in the various assays and the operators for the different assays used in the study were blinded to the identity of the samples.

Reporting for specific materials, systems and methods

We require information from authors about some types of materials, experimental systems and methods used in many studies. Here, indicate whether each material, system or method listed is relevant to your study. If you are not sure if a list item applies to your research, read the appropriate section before selecting a response.

Materials & experimental systems

n/a	Involved in the study	
☐	☒	Antibodies
☐	☒	Eukaryotic cell lines
☒	☐	Palaeontology and archaeology
☐	☒	Animals and other organisms
☒	☐	Clinical data
☒	☐	Dual use research of concern

Methods

n/a	Involved in the study	
☒	☐	ChIP-seq
☐	☒	Flow cytometry
☒	☐	MRI-based neuroimaging

Antibodies

Antibodies used	Anti-Human Perforin -PerCP-eFluor710 (clone deltaG9), eBiosciences (Catalog# 46-994-42)<br>Anti-human CD159a-PE (NKG2A; clone Z199), Beckman Coulter (Catalog# A60797)<br>Anti-human CD3-Cy-7APC (clone SP34-2), BD Biosciences (Catalog# 557749)

Anti-human CD4-APC (clone RPA-T4), BD Biosciences (Catalog# 555349)
Anti-human CD8-Alexa700 (clone RPA-T8), BD Biosciences (Catalog# 561453)
Anti-human CD95-FITC (clone DX2), BD Biosciences (Catalog# 556640)
Anti-human CD28-Cy-5PE (clone CD28.2), BD Biosciences (Catalog# 561791)
Anti-human CD20-Pacific Blue (clone 2H7), Biologend (Catalog# 302320)
Anti-human KIR2D-PE (clone NKVFS1), Miltenyi Biotech (Catalog# 130-092-688)
Anti-human Ki-67-FITC (clone B56), BD Biosciences (Catalog# 665127).
Anti-humanIL-2-PE (clone MQ1-17H12), BD Biosciences (Catalog# 554566)
Anti-human IFN-γ-FITC (clone B27), BD Biosciences (Catalog# 552887)
Anti-humanTNF-α-Cy7PE (clone Mab11), BD Biosciences (Catalog# 557647)
Anti-human CD14--Pacific Blue (Clone M5E2), BD Biosciences (Catalog# 558121)

Validation

All antibodies used were titrated using rhesus macaque PBMC and have been used extensively in earlier studies (Eberly et al J Immunol 2009, 182(3): 1439 -48, Moore AC J. Virol 2012, 86(2): 1069 - 78, Mattapallil et al Nature 2005, 434(7037): 1093 - 7.

Eukaryotic cell lines

Policy information about cell lines and Sex and Gender in Research

Cell line source(s)

TZM-bl cells were obtained from the NIH AIDS Research and Reference Reagent Program

Authentication

The cell line was obtained through the NIH HIV Reagent Program, Division of AIDS, NIAID, NIH: TZM-bl Cells, ARP-8129, contributed by Dr. John C. Kappes, Dr. Xiaoyun Wu and Tranzyme Inc., and has been extensively published (J. Virol. 74 (2002): 8358-67; J. Virol. 72 (1998), 2855-64; J. Virol. 83 (2009): 8289-92; Antimicrob. Agents Chemother. 46 (2002): 1896-905; Nat. Commun. 12 (2021): 2257). Cells are maintained by the laboratory's dedicated cell culture core with strict standard operating procedures in place for thawing, expansion, maintenance and usage of cells. At receipt, the cell line was expanded and cell banks were established. To ensure cell integrity and optimal performance, co-receptor (CD4, CCR5 and CXCR4) expression levels and cell infectability were monitored over time to establish guidelines for allowable cell passage number and culture duration. Cell morphology and viability is carefully monitored during use. All work is conducted in compliance with Good Clinical Laboratory Practices (GCLP).

Mycoplasma contamination

No mycoplasma contamination was detected.

Commonly misidentified lines
(See ICLAC register)

No misidentified cell lines were used.

Animals and other research organisms

Policy information about studies involving animals; ARRIVE guidelines recommended for reporting animal research, and Sex and Gender in Research

Laboratory animals

Thirteen (4 - 7 years old) Mamu A*01– and B17– rhesus macaques of Indian origin (unvaccinated control; n = 6 and vaccinated; n = 7) that were seronegative for Vaccinia virus (determined in the laboratory of Dr. Rama Amara at Emory University), SIV, simian retrovirus (SRV), Herpes-B and simian T-cell leukemia virus (STLV) type-1 were used in this study.

Wild animals

No wild animals were used.

Reporting on sex

Only male animals were used in this study.

Field-collected samples

No field samples were collected.

Ethics oversight

The animals were housed at Bioqual Inc., in accordance with the American Association for Accreditation of Laboratory Animal guidelines at Bioqual, Inc. (Kensington, MD), and all the procedures were performed according to protocol approved by the Institutional Animal Use and Care Committee at Bioqual and accepted by the Uniformed Services University of the Health Sciences.

Note that full information on the approval of the study protocol must also be provided in the manuscript.

Flow Cytometry

Plots

Confirm that:

☒ The axis labels state the marker and fluorochrome used (e.g. CD4-FITC).

☒ The axis scales are clearly visible. Include numbers along axes only for bottom left plot of group (a 'group' is an analysis of identical markers).

☒ All plots are contour plots with outliers or pseudocolor plots.

☒ A numerical value for number of cells or percentage (with statistics) is provided.

Methodology

Sample preparation	PBMC were surface labeled with a panel of anti-CD3, CD20, CD14, NKG2A, KIR2D antibodies. After the cells were fixed and permeabilized, they were labeled with anti-perforin and Ki-67 antibodies. Labeled cells were fixed with 0.5% paraformaldehyde and analyzed using an LSR II flow cytometer (BD Biosciences)
Instrument	BD LSR II
Software	Flow Jo Software 9.0
Cell population abundance	A total of 1 million total events were collected where possible for analysis. Gating trees were set to examine the desired populations whose abundance was dependent on the markers used.
Gating strategy	PBMC were surface labeled with a panel of anti-CD3, CD20, CD14, NKG2A and KIR2D antibodies. After the cells were fixed and permeabilized, they were labeled with anti-perforin and Ki-67 antibodies. Labeled cells were fixed with 0.5% paraformaldehyde and analyzed using an LSR II flow cytometer (BD Biosciences). Frequencies of NK cell subsets within each CD3-CD20-CD14-NKG2A+ NK cell subset that expressed Ki67, KIR2D and Perforin were determined using Boolean gating using Flowjo.

☒ Tick this box to confirm that a figure exemplifying the gating strategy is provided in the Supplementary Information.

This checklist template is licensed under a Creative Commons Attribution 4.0 International License, which permits use, sharing, adaptation, distribution and reproduction in any medium or format, as long as you give appropriate credit to the original author(s) and the source, provide a link to the Creative Commons license, and indicate if changes were made. The images or other third party material in this article are included in the article's Creative Commons license, unless indicated otherwise in a credit line to the material. If material is not included in the article's Creative Commons license and your intended use is not permitted by statutory regulation or exceeds the permitted use, you will need to obtain permission directly from the copyright holder. To view a copy of this license, visit http://creativecommons.org/licenses/by/4.0/
